# Completeness and consistency of primary outcome reporting in COVID-19 publications in the early pandemic phase: a descriptive study

**DOI:** 10.1186/s12874-023-01991-9

**Published:** 2023-07-29

**Authors:** Marlene Stoll, Saskia Lindner, Bernd Marquardt, Maia Salholz-Hillel, Nicholas J. DeVito, David Klemperer, Klaus Lieb

**Affiliations:** 1 Department of Psychiatry and Psychotherapy, University Medical Center of the Johannes Gutenberg University Mainz, Mainz, Germany; 2 Leibniz Institute for Resilience Research (LIR), Mainz, Germany; 3 QUEST Center for Responsible Research, Berlin Institute of Health (BIH), Charité Universitätsmedizin Berlin, Berlin, Germany; 4 Bennett Institute for Applied Data Science, Nuffield Department of Primary Care Health Sciences, University of Oxford, Oxford, UK; 5 Ostbayrische Technische Hochschule Regensburg, Regensburg, Germany

**Keywords:** Primary outcome, Discrepancy, Reporting quality, Methodological quality, COVID-19, Trial registry

## Abstract

**Background:**

The COVID-19 pandemic saw a steep increase in the number of rapidly published scientific studies, especially early in the pandemic. Some have suggested COVID-19 trial reporting is of lower quality than typical reports, but there is limited evidence for this in terms of primary outcome reporting. The objective of this study was to assess the prevalence of completely defined primary outcomes reported in registry entries, preprints, and journal articles, and to assess consistent primary outcome reporting between these sources.

**Methods:**

This is a descriptive study of a cohort of registered interventional clinical trials for the treatment and prevention of COVID-19, drawn from the DIssemination of REgistered COVID-19 Clinical Trials (DIRECCT) study dataset. The main outcomes are: 1) Prevalence of complete primary outcome reporting; 2) Prevalence of consistent primary outcome reporting between registry entry and preprint as well as registry entry and journal article pairs.

**Results:**

We analyzed 87 trials with 116 corresponding publications (87 registry entries, 53 preprints and 63 journal articles). All primary outcomes were completely defined in 47/87 (54%) registry entries, 31/53 (58%) preprints and 44/63 (70%) journal articles. All primary outcomes were consistently reported in 13/53 (25%) registry-preprint pairs and 27/63 (43%) registry-journal article pairs. No primary outcome was specified in 13/53 (25%) preprints and 8/63 (13%) journal articles. In this sample, complete primary outcome reporting occurred more frequently in trials with vs. without involvement of pharmaceutical companies (76% vs. 45%), and in RCTs vs. other study designs (68% vs. 49%). The same pattern was observed for consistent primary outcome reporting (with vs. without pharma: 56% vs. 12%, RCT vs. other: 43% vs. 22%).

**Conclusions:**

In COVID-19 trials in the early phase of the pandemic, all primary outcomes were completely defined in 54%, 58%, and 70% of registry entries, preprints and journal articles, respectively. Only 25% of preprints and 43% of journal articles reported primary outcomes consistent with registry entries.

**Supplementary Information:**

The online version contains supplementary material available at 10.1186/s12874-023-01991-9.

## Introduction

The COVID-19 pandemic has led to an increase in the number of rapidly published scientific studies [1–5] and to changes in the study publication process. Preprints have increased substantially [6, 7] and the peer review process has been altered [3], especially at the beginning of the pandemic. This may have impacted the quality of COVID-19 publications [1, 4, 5, 8].

Official registries for trial registration have been established to increase transparency and to make it possible to determine whether reports of clinical trials have been modified or whether the results have not been published at all [9–14]. Published results should be consistent with the trial registration information and any deviations should be noted [10, 15, 16]. To ensure the replicability and traceability of results, primary outcomes should be completely defined across all information sources, i.e., sufficient information should be provided so that others can use the same primary outcomes [16–20]. This also contributes to the sustainability of the scientific finding because evidence from completely reported primary outcomes can be included in evidence syntheses (e.g., in the form of a meta-analysis) and thus contribute to evidence-based medicine and to long-term knowledge gain [21].

Prior to the pandemic, trials often were registered with inadequately defined primary outcomes [15, 16, 22]. Primary outcomes were changed after study completion [23], and many primary outcomes were inexplicitly switched or reported poorly or not at all in subsequent publications [10, 18, 24–28]. These inconsistencies were commonly reported in the literature. However, previously published studies showed considerable variation in their prevalence [10, 15, 22, 26, 28–36]. Factors that could negatively influence the quality of reporting are authors’ conflicts of interest, study funding source, author characteristics, journal characteristics and study design [10, 22, 23, 33, 34, 37, 38]. Prior work has shown little difference in reporting quality between preprints and journal articles [39, 40]. The quality of COVID-19 trial registration data has been shown to vary [12], and discrepancies have been noted between preprints and journal articles of clinical studies [7, 41].

This study’s objective was to complement the current research on the quality of COVID-19 studies by providing evidence on primary outcome reporting quality early in the pandemic. Specifically, we assessed 1) the prevalence of completely defined primary outcomes in registry entries, preprints and journal articles and 2) the prevalence of consistent primary outcome reporting between registry entry and preprint, and between registry entry and journal article pairs. We also explored the impact of publication type, registration time point, study design, and involvement of pharmaceutical companies on primary outcome completeness and consistency.

## Methods

### Study sample

We evaluated a cohort of registered interventional clinical trials for the treatment and prevention of COVID-19 with full or interim results published as either preprints or journal articles. We drew our sample from the open access DIssemination of REgistered COVID-19 Clinical Trials (DIRECCT) dataset, previously described in Salholz-Hillel et al. (2021) [42]. The dataset included 3749 COVID-19 trials sourced from the International Clinical Trials Registry Platform (ICTRP) list of registered COVID-19 studies and individual clinical trial registries which were completed by June 30, 2020. The dataset also included 155 results identified via automated and manual searches in the COVID-19 Open Research Dataset (CORD-19), PubMed, EuropePMC, Google Scholar, Google, and registries between 30 June 2020 and 18 January 2021 [42].

**Fig. 1:**
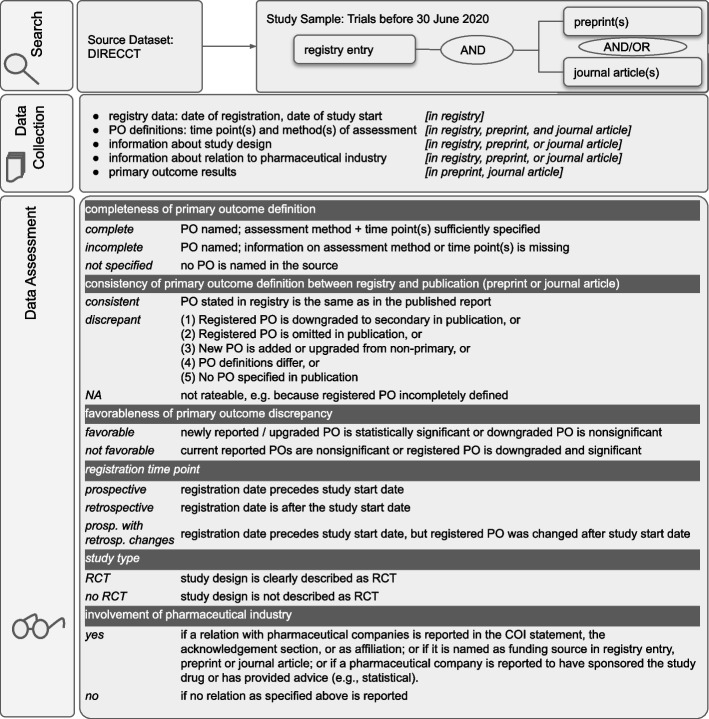
Methodological approach of this study. PO = primary outcome; prosp. = prospective; retrosp. = retrospective

For our analysis, we screened all results for publication type and included trials with full or interim results, published as preprints or journal articles and which were available in full text and in English language. We excluded summary results and other results types (e.g., grey literature, research letters). If there was more than one registry entry per trial, we included the registry entry that provided the most detailed information on primary outcomes, e.g., the registry entry that, in case of a registry entry update, provided information on both current and originally registered primary outcome measures. If there was more than one preprint or journal article, all were assessed. Each trial assessment therefore included the assessment of exactly one registry entry and one or more preprints and/or one or more journal articles (Fig. 1). Trial selection was performed by 2 researchers (MS, SL) and discussed in case of disagreements. Data and code for trial selection is available online [43].

### Data collection process

Data was collected from registry entries, preprints and journal articles. Two researchers independently coded each evidence source (i.e., registry entry, preprint, or journal article). Deviations were solved in discussion with a third researcher. Extraction and reconciliation was performed in Google Sheets. The data extraction and assessment process is depicted in Fig. 1 and described in detail in Additional file 1.

### Data items

Data assessment was performed by two researchers and discussed with a third researcher in case of disagreement (MS, SL, BM). Definitions of all variables are depicted in Fig. 1. The two main outcomes of this study are the prevalence of complete primary outcome reporting and the prevalence of consistent primary outcome reporting. Assessment of primary outcome completeness was based on CONSORT 2010 [19]. A primary outcome was thus rated *complete* if its definition included the “how” (measure and metric) and “when” (time point or time frame) of its assessment [18, 19]. At the evidence source level, we distinguished whether all primary outcomes in the source were completely defined or whether at least one primary outcome was incompletely defined.

Consistency in primary outcome reporting was assessed according to a slightly modified classification following Mathieu et al. (2009) [16] (see Fig. 1 and Additional file 1). Only the registered primary outcomes that were completely defined were used to assess primary outcome consistency. In case of a discrepancy, we additionally checked whether authors justified the deviation from the registry entry in the publication, as CONSORT allows. Researchers agreed on 92% and 93% of the completeness and consistency ratings, respectively.

We further assessed the trial’s study design, involvement of pharmaceutical companies, and registration time point (Fig. 1). If a primary outcome in a prospective registry entry was changed retrospectively, trials’ registration time point was considered to be *prospectively registered with retrospective changes*. The latest version of the primary outcome available in the registry was used for the assessment of primary outcome consistency. For each completely defined primary outcome we assessed statistical significance by checking whether *p* values and/or 95% confidence intervals indicated *p* < 0.05 (or, if applicable, at the otherwise specified alpha level). Additionally, primary outcome discrepancies were assessed as to whether they favored study results [15, 16, 44, 45]: a discrepancy was considered to be favorable if a newly added primary outcome favored the study results (e.g., statistical significance) or a downgraded primary outcome did not favor the study results (e.g., nonsignificance).

### Data synthesis

Data were summarized by calculating absolute and relative frequencies. The analyzed sample consisted of all COVID-19 clinical trials that were completed in the early phase of the COVID-19-pandemic (by June 30, 2020) with results published in the form of a preprint or a journal article by January 18, 2021. The investigated trials represent the entire population of trials that match these inclusion criteria. Since our aim was to describe this population rather than testing assumptions on a larger population, no inferential statistics are reported. The study has not been preregistered. The study report was written according to guidelines for reporting meta-epidemiological methodology research [46].

## Results

### Study selection

**Fig. 2:**
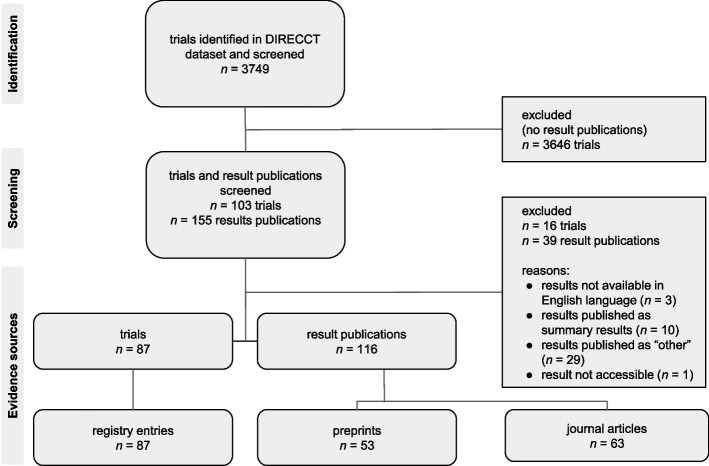
Flowchart depicting study selection and investigated evidence sources. Evidence source = registry entry, preprint or journal article; PO = primary outcome; published as “other” = results are published in different ways than in preprints or journal articles, e.g., in grey literature or research letters

From the 3749 trials in the source dataset, 103 trials with 155 result publications were screened (Fig. 2). Our final analysis dataset comprised 87 registry entries, 53 preprints and 63 journal articles (see Additional file 2). We analyzed 53 registry-preprint pairs, 63 registry-journal article pairs, and 18 registry-preprint-journal triplets.

### Study characteristics

Most of the trials in our sample were registered in the ClinicalTrials.gov registry (51%, 44/87) and the Chinese Clinical Trial registry (34%, 30/87). In 19/87 (22%) registry entries, changes were made to the originally registered primary outcome. Nine of these 19 cases (47%) were retrospective changes made to a prospective registration. Eight of the 19 changes (42%) were justified in the publication.

**Fig. 3:**
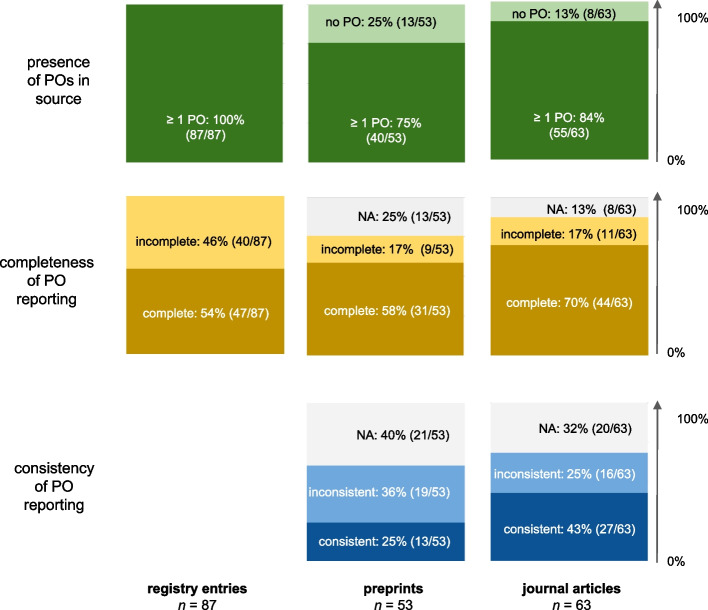
Primary outcome presence, completeness and consistency on evidence source level. All frequencies are given at evidence source level. incomplete: ≥ 1 PO in evidence source is incompletely defined; inconsistent: ≥ 1 PO in evidence source is inconsistently reported; PO = primary outcome

All 87/87 (100%) registries contained a primary outcome, but 13/53 (25%) preprints and 8/63 (13%) journal articles did not indicate a specific primary outcome (Fig. 3). The number of primary outcomes per source ranged between 1–16 in registry entries, 0–5 in preprints, and 0–4 in journal articles.

### Unit of analysis: primary outcomes

In total, we found 282 primary outcomes across all sources. All primary outcomes and their ratings can be found in Additional file 3. Overall 170/282 (60%) were completely defined (i.e., method and time point(s) of assessment were mentioned, per CONSORT) and 112/282 (40%) were incompletely defined. The relative frequency of completely defined primary outcomes was highest for primary outcomes in journal articles (58/69, 84%) and lowest for primary outcomes in registry entries (71/162, 44%).

**Fig. 4:**
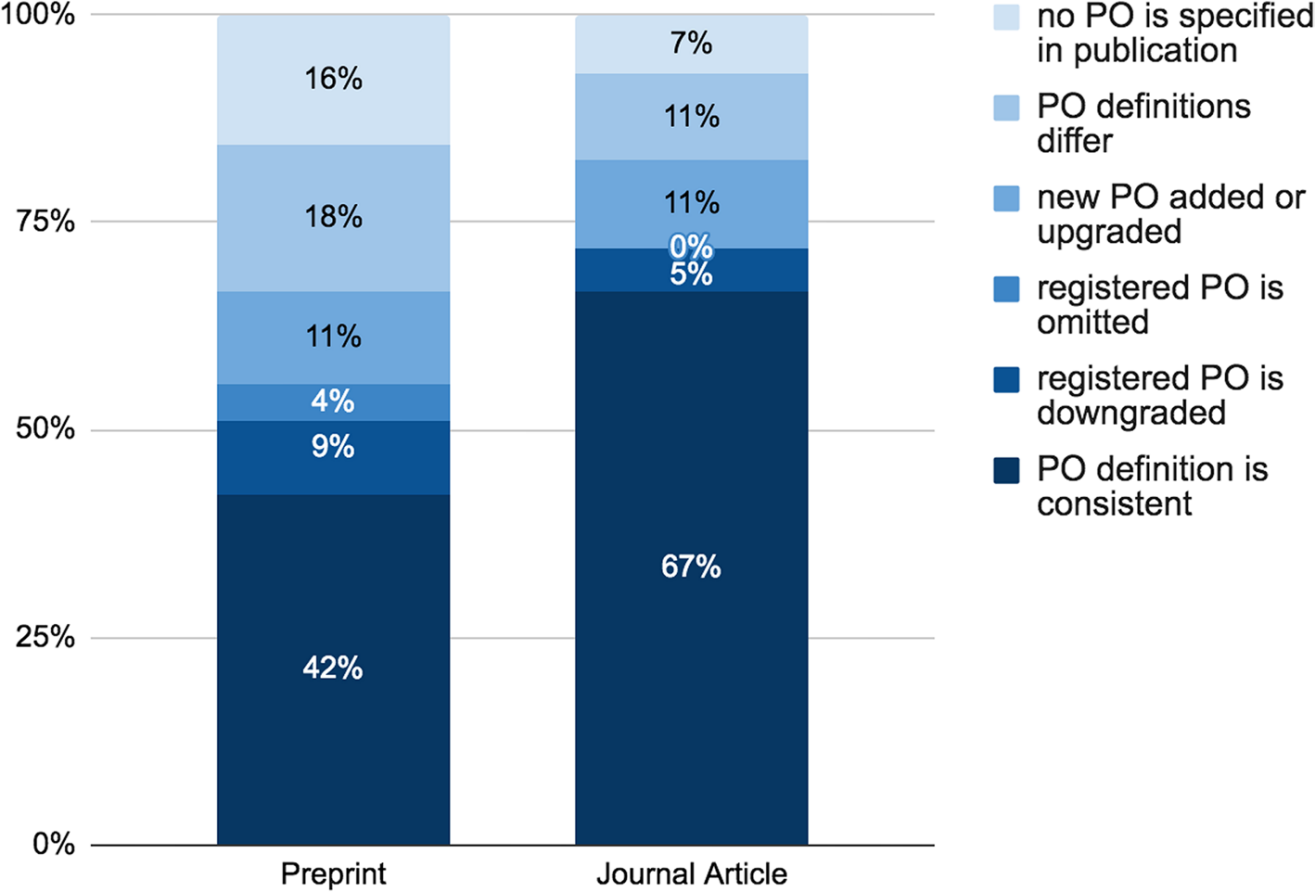
Primary outcome consistency on primary outcome level. Different shades of blue represent different types of discrepancies; reference number for 100% are all cases of primary outcome consistency assessment in registry-preprint pairs (*n* = 45) and in registry-journal article pairs (*n* = 57) on primary outcome level. PO = primary outcome

We were able to make 102 primary outcome consistency assessments based on completely defined registered primary outcomes. In 57/102 (56%) cases, primary outcome reporting was consistent between the registered and the published primary outcome, while in 45/102 (44%), a discrepancy was found. None of these were justified by the study authors. The most common discrepancy was that primary outcome definitions differed (14/45, 31%). Frequencies of the different types of discrepancies are shown in Fig. 4.

Of the 57 consistent cases, 8 (14%) reported a statistically significant result for the primary outcome, 31 (54%) reported a nonsignificant result and 18 (32%) were not rateable (e.g., published primary outcome was incompletely defined). Of the 45 discrepant cases, 2 (4%) reported a statistically significant result for the primary outcome, 8 (18%) reported a nonsignificant result, and 35 (78%) were not rateable. We furthermore investigated the favorableness of primary outcome discrepancy. Of the 45 discrepant cases, 8 (18%) were rated as favorable (i.e., the discrepancy was in favor of the study results) and 8 (18%) were rated as non-favorable. In the other 29 (64%) cases, we were not able to assess whether the discrepancy favored study results because information was missing.

### Unit of analysis: whole evidence source

We then examined the whole evidence source (registry entry, preprint or journal article). Complete primary outcome reporting was found in 122/203 (60%) sources, whereas in 60/203 (30%) at least one primary outcome was incompletely defined; in 21/203 (10%) no primary outcome was specified. The relative frequency of sources with all primary outcomes being completely defined was highest for journal articles (44/63, 70%) and lowest for registry entries (47/87, 54%); compared to 31/53 (58%) for preprints (Fig. 3).

We found 116 evidence source pairs with primary outcome reporting. Of these, 41/116 (35%) had one or more incompletely registered primary outcome, and therefore consistency could not be assessed. Of the 75/116 (65%) evidence source pairs with completely defined registered primary outcomes, consistent primary outcome reporting was found in 40/75 (53%), while 35/75 (47%) showed at least one discrepancy. The relative frequency of consistent primary outcome reporting was higher in registry-journal article pairs (27/63, 43%) than in registry-preprint pairs (13/53, 25%) (Fig. 3).

The absolute and relative frequencies of complete as well as consistent primary outcome reporting based on type of evidence source, registration time point, study design, and involvement of pharmaceutical companies are shown in Table 1. Of all 87 trials, 53 (61%) were registered retrospectively, 25 (29%) prospectively, and 9 (10%) were registered prospectively with retrospective changes; 60% (52/87) were RCTs, and 49% (43/87) reported some involvement of pharmaceutical companies.

**Table 1:** Contingency tables for primary outcome completeness and consistency on evidence source level

		**registry entries (** ***n*** ** = 87)**				**preprints (** ***n*** ** = 53)**				**journal articles (** ***n*** ** = 63)**			
**completeness**		**n**_**0**_	**all POs completely defined** **n (%)**	≥ **1 PO** **incompletely defined** **n (%)**	**no PO specified** **n (%)**	**n**_**0**_	**all POs completely defined** **n (%)**	≥ **1 PO** **incompletely defined** **n (%)**	**no PO specified** **n (%)**	**n**_**0**_	**all POs completely defined** **n (%)**	≥ **1 PO** **incompletely defined** **n (%)**	**no PO specified** **n (%)**
total		87	47 (54)	40 (46)	0 (0)	53	31 (58)	9 (17)	13 (25)	63	44 (70)	11 (17)	8 (13)
registration time point	retrosp.	53	25 (47)	28 (53)	0 (0)	32	20 (63)	5 (16)	7 (22)	39	27 (69)	6 (15)	6 (15)
	prosp.	25	13 (52)	12 (48)	0 (0)	14	5 (36)	3 (21)	6 (43)	18	12 (67)	5 (28)	1 (6)
	changes	9	9 (100)	0 (0)	0 (0)	7	6 (86)	1 (14)	0 (0)	6	5 (83)	0 (0)	1 (17)
study design	no RCT	35	19 (54)	16 (46)	0 (0)	26	11 (42)	4 (15)	11 (42)	21	10 (48)	5 (24)	6 (29)
	RCT	52	28 (54)	24 (46)	0 (0)	27	20 (74)	5 (19)	2 (7)	42	34 (81)	6 (14)	2 (5)
pharma	yes	43	30 (70)	13 (30)	0 (0)	21	16 (76)	1 (5)	4 (19)	38	31 (82)	6 (16)	1 (3)
	no	44	17 (39)	27 (61)	0 (0)	32	15 (47)	8 (25)	9 (28)	25	13 (52)	5 (20)	7 (28)
**consistency**						**n**_**0**_	**all POs consistent** **n (%)**	≥ **1 PO inconsistent** **n (%)**	**NA**^**a**^ **n (%)**	**n**_**0**_	**all POs consistent** **n (%)**	≥ **1 PO inconsistent** **n (%)**	**NA**^**a**^ **n (%)**
total		-				53	13 (25)	19 (36)	21 (40)	63	27 (43)	16 (25)	20 (32)
registration time point	retrosp.	-				33	7 (21)	11 (33)	15 (45)	39	15 (38)	12 (31)	12 (31)
	prosp.	-				13	1 (8)	6 (46)	6 (46)	18	7 (39)	3 (17)	8 (44)
	changes	-				7	5 (71)	2 (29)	0 (0)	6	5 (83)	1 (17)	0
study design	no RCT	-				25	4 (16)	13 (52)	8 (32)	21	6 (29)	7 (33)	8 (38)
	RCT	-				28	9 (32)	6 (21)	13 (46)	42	21 (50)	9 (21)	12 (29)
pharma	yes	-				21	9 (43)	6 (29)	6 (29)	38	24 (63)	10 (26)	4 (11)
	no	-				32	4 (13)	13 (41)	15 (47)	25	3 (12)	6 (24)	16 (64)

Some numbers in columns do not add up to 100 because of rounding; *PO* Primary outcome, *retrosp* Retrospective, *prosp* Prospective, *changes* Prospective registration with retrospective changes, *RCT* Randomized controlled trial, *pharma* Involvement of pharmaceutical industry

^a^registered primary outcome incompletely defined, therefore not eligible for primary outcome consistency assessment

Complete primary outcome reporting occured more frequently in evidence sources where involvement of pharmaceutical companies was reported vs. in sources where it was not reported (76% (77/102) vs. 45% (45/101)) and in RCTs vs. other study designs (68% (82/121) vs. 49% (40/82)). Consistent primary outcome reporting also occurred more frequently in evidence sources where involvement of pharmaceutical companies was reported vs. in sources where it was not reported (56% (33/59) vs. 12% (7/57)) and it was more frequent in RCTs vs. other study designs (43% (30/70) vs. 22% (10/46)).

### Primary outcome consistency across registry-preprint-journal article triplet

We found 18/87 (21%) trials with at least one primary outcome reported across all three evidence sources. Of these 18 trials, 10 (56%) trials had a completely defined registered primary outcome and were further investigated.

In 5/10 (50%) trials with completely defined registered primary outcomes and results being published in both preprints and journal articles, primary outcome definitions were consistent across all sources. In 2/10 (20%) of these trials, primary outcomes were mentioned in the registry only. One trial (1/10, 10%) had a primary outcome specified in just the journal article (and none in the preprint), but the definition in the journal article differed from the registry entry. Another trial (1/10, 10%) showed discrepancies across all three sources, and in yet another (1/10, 10%), the registered primary outcome was downgraded in both preprint and journal article while preprint and journal article reported consistent primary outcomes.

## Discussion

### Summary of evidence

In a set of registered interventional clinical trials for the treatment and prevention of COVID-19 and the publications of their results, complete primary outcome reporting was more frequent in journal articles than in preprints and registry entries, and consistent primary outcome reporting was more frequent in registry-journal article pairs than in registry-preprint pairs. We further found that trials in our sample which reported the involvement of pharmaceutical companies (e.g. funding, sponsoring or consultative role) more frequently showed complete and consistent primary outcome reporting than trials in which no such involvement was reported. RCTs also more frequently showed complete and consistent primary outcome reporting than trials with other study designs.

### Findings in context

Our results are in line with previous findings that COVID-19 registry entries and studies in the early phase of the pandemic showed low reporting quality [1, 47]. The findings are further in line with research that found changes in study results or abstract conclusions between and within preprints and journal articles reporting COVID-19 trials [7]. COVID-19 studies also frequently had outcomes missing in either preprint or journal articles [41]. Our study adds a detailed analysis of primary outcome reporting across all three evidence sources. Additionally, we provide explorative findings for the role of registration time point, involvement of pharmaceutical companies, and study design.

Our results reflect the findings of Li et al. (2018) showing high prevalence of inconsistent outcome reporting in non-COVID-19 research [34]. Although several previous studies have assessed completeness and consistency of primary outcome reporting, comparisons between studies are difficult due to diversity of trial samples [16, 29] and heterogeneity of methodological approaches [32].

Our finding of better reporting quality of RCTs and trials in which pharmaceutical companies were involved reflects previous work that shows trials involving pharmaceutical companies have slightly better reporting quality of study methods [22, 36, 38, 48, 49]. RCTs are considered the gold standard of clinical trial design and may be accompanied by generally higher demands on methodology and reporting quality.

Registry entries always contained at least one primary outcome, while 13 preprints and 8 journal articles did not define any primary outcomes. Structured and required fields for primary outcomes in registries promote their inclusion by default, while this is not the case for preprints and journal articles. In preprints, no quality review takes place, while in journal articles, editors or reviewers might cross-check compliance with reporting standards but do not necessarily do so [50, 51]. Our findings indicate that at least in those 8 journal articles that did not define any primary outcomes, the review process has not included these checks. At the same time, the publication process and the work of the editor and reviewers may have some effect on better reporting practices as the prevalence for complete and consistent primary outcome reporting in this analysis was highest for journal articles.

### Strengths and limitations

A strength of our approach is that we investigated the entire population of registered COVID-19 trials early in the pandemic, thus the reported prevalence is not an estimation but a measurement. Moreover, we provide detailed information on the completeness and consistency of primary outcomes across three different sources: registry entry, preprint and journal article. There are also limitations: First, we did not include trial protocols, which are another source of key trial information; however, there is no guarantee these will be made publicly available in the same way a registry entry is. Second, as only 18 trials had information available in a registry, preprint, and journal publication, our study provides between- rather than within-comparisons. Third, we only investigated primary outcome discrepancies as well as its favorableness if sufficient information was available. As we only investigated discrepancies if primary outcomes were completely defined in the registry and only 54% of the registered primary outcomes were completely defined, we may have underestimated the prevalence of primary outcome inconsistency. Also, we were not able to assess whether a discrepancy was in favor of the study if there was no information on the results of this outcome (e.g., if a registered primary outcome is omitted from the published article).

### Implications of the findings

We observed eight cases (18%) where the discrepancy favored the trial’s results (e.g., a newly added primary outcome was statistically significant) and eight where it did not (18%), while most (64%) were not rateable due to lack of information. Looking at statistical significance of primary outcomes alone, we observed that only 4% of discrepant cases reported statistically significant results compared to 14% of consistent cases. At the same time, not rateable cases were more frequent in the discrepant cases (78%) than in the consistent cases (32%). This suggests that the discrepancies may be due to negligence or error, rather than intent to make the study results more favorable. The hurry to publish and to gain evidence on COVID-19, especially at the beginning of the pandemic, might have further increased these reporting issues. This underlines the importance for standardized reporting of primary outcomes in registries, preprints and journal articles. Only if study methods and results are reported in a complete, standardized way, and reliably checked for completeness, can they be fully understood. Transparency of study methods is a basic requirement for the reduction and assessment of bias in research [9–14].

Our findings indicate a high prevalence of incomplete and inconsistent primary outcome reporting practices. Reporting guidelines for registrations (e.g., WHO International Standards for Clinical Trial Registries [20]), and publications (e.g., CONSORT [19]) should be rigorously adhered to, to help ensure high quality research [50]. The recent CONSORT-2020 extension added recommendations for outcome-specific information [52]. Use and implementation of these guidelines by authors, editors, and reviewers could contribute to improved outcome reporting quality in future [21].

Our results show higher reporting quality in journal articles, which indicates that factors inherent in the publication process such as peer review may already contribute to a better reporting quality to some extent. Nevertheless, researchers still need to be made aware of and recognize the importance of complete and consistent reporting practices [51]. Our findings in context with similar studies emphasize the importance of guidelines and/or checklists for authors, reviewers, and editors to facilitate an easy and comparable assessment of primary outcome reporting quality [34].

## Conclusions

In this investigation of COVID-19 trial publications early in the pandemic, 54%, 58%, and 70% of registry entries, preprints and journal articles, respectively, reported all primary outcomes completely defined. A considerable number of publications (25% of preprints and 13% of journal articles) did not specify any primary outcome. Only in 25% and 43% of preprints and journal articles, respectively, were primary outcomes consistently reported with the entries in the registry. Since we investigated the entire population of COVID-19 trials in this early phase of the pandemic through June 30, 2020, we can conclude that complete and consistent primary outcome reporting was more prevalent in journal articles than in preprints, indicating the importance of the peer review process. Despite the need for rapid evidence dissemination and pressure for expedited publication, quality of primary outcome reporting should not suffer and study authors, editors and reviewers should pay particular attention to reporting quality in such exceptional times.

## Supplementary Information

**Additional file 1: **Detailed report of data collection process, data items and study outcomes.

**Additional file 2: Table 1. **Sample from the open access DIssemination of REgistered COVID-19 Clinical Trials (DIRECCT) database. 

**Additional file 3: Table 2. **Primary outcome definitions in registry, preprint and journal articles and rating of completeness and discrepancies in the 87 included trials.

## Data Availability

The dataset supporting the conclusions of this article is included within the article and its additional files.
