# Supplementary material for: Completeness and consistency of primary outcome reporting in COVID-19 publications in the early pandemic phase: a descriptive study
Source: BMC Med Res Methodol. 2023 Jul 29;23:173. doi: 10.1186/s12874-023-01991-9 (PMC10385884; doi:10.1186/s12874-023-01991-9)
Supplement: Supplementary file 1 — Additional file 1: Detailed report of data collection process, data items and study outcomes. [file 12874_2023_1991_MOESM1_ESM.pdf]

Additional File 1: Detailed report of data collection process, data items and study outcomes

Data collection process

Two researchers independently coded each evidence source (i.e., registry entry, preprint, or journal article). Deviations were solved in discussion with a third researcher (MS, SL and BM). Extraction and reconciliation was performed in Google Sheets.

We screened all evidence sources for information on primary outcomes by searching for the terms “primary outcome(s)”, “primary endpoint(s)”, “main outcome(s)”, and “main endpoint(s)”. If a primary outcome was explicitly named, we searched for specification of method and time point / time frame of primary outcome assessment in the whole text. If no outcome was reported as being primary, we classified the object as *no primary outcome specified*. In order to take into account the possibility that primary outcomes in registry entries can be changed over time, we analyzed the “history of changes” of the registry entries. We checked whether the changes in the registry entry were verified in the publication and considered this accordingly in the investigation of primary outcome consistency. For the analysis of primary outcome completeness, we restricted ourselves to the newest submitted version of the primary outcome. Outcomes were not considered to have been changed if there were only minor differences between original and changed versions that do not impact the definition of the primary outcome (e.g., correction of spelling errors). To investigate the favorableness of results, information on primary outcome results were only extracted from preprints and journal articles of study type “RCT” and with completely defined primary outcomes.

We also searched all evidence sources for information on study type to decide whether the trial was an RCT or not and for information on involvement of pharmaceutical companies. We screened conflict of interest statements, acknowledgement sections, author affiliations and, in preprints and journal articles, searched for the terms “provided”, “sponsored”, "provision", "gift", “donation”. We also screened the funding source section of registry entries, preprints and journal articles.

Data items and study outcomes

After data collection, we assessed the variables specified below. All assessments were performed by two independent researchers and discussed with a third researcher in case of disagreement (MS, SL, and BM).

Completeness of primary outcome definition: A primary outcome was considered to be *completely defined* if the primary outcome (or synonyms, as explained above) was named and assessment method as well as time point(s) or time frame of assessment were specified in a way that allows others to use the same outcomes (CONSORT 2010). It was considered to be *incompletely defined* if the primary outcome was named and information on the method or time point/time frame of measurement was missing. If no primary outcome was named, this was rated as *no primary outcome specified*. We investigated primary outcomes completeness by two units of analysis: the primary outcome (completely defined vs. incompletely defined) and the whole investigation object (all primary outcomes in the entry/publication are completely defined vs. at least one primary outcome in the entry/publication is incompletely defined).

Consistency of primary outcome definition: We investigated consistency in primary outcome reporting according to a slightly modified classification of Mathieu et al. (2009) who refers to Chan et al. (2004). Unlike Mathieu we did not assess whether the published primary outcome was registered as secondary outcome since we did not investigate registered secondary outcomes. We additionally classified the case when a primary outcome was registered but none was specified in the publication. We further did not only check whether timing of assessment of the primary outcomes differed but also whether method of assessment differed. As Mathieu et al. (2009), we compared primary outcomes between registry entries and publications only for trials with completely defined primary outcomes in the registry entry. Each comparison was classified as one of the following types of discrepancy:

- *Primary outcome definition is consistent*: The primary outcome in the registry entry is the same as in the published report (i.e., preprint or journal article).
- *Registered primary outcome is downgraded*: The primary outcome stated in the trial registry is downgraded to a secondary outcome in the publication.
- *Registered primary outcome is omitted*: The primary outcome stated in the registry entry is omitted from the publication.
- *New primary outcome is added or upgraded*: A new primary outcome not stated as primary outcome in the registry entry is included in the published report or upgraded from non-primary.
- *Primary outcome definitions differ*: The method and/or timing of assessment of the registered and published primary outcomes differ but refer to the same variable.
- *No primary outcome is specified in publication*: In the publication, no primary outcome is clearly specified.

Since an evidence source could have more than one primary outcome, more than one type of discrepancy per trial was possible. In case primary outcomes in the registry entry had been changed, so that an original (outdated) as well as a current version of a registered primary outcome was available, we compared the current version to the published primary outcome. Additionally, we looked in all trials whether new primary outcomes were published that had not been registered as primary outcome (e.g., a registered secondary outcome that becomes primary in the article or an outcome that does not appear at all in the registry but is introduced as primary in the article).

Consistency of primary outcome reporting was investigated by two units of analysis: First, the consistency of the primary outcome and second, of the whole evidence source (all primary outcomes in the publication consistent with the registry entry vs. at least one primary outcome in the publication is somehow discrepant from the registry entry).

Favorableness of primary outcome discrepancy: Primary outcome discrepancies were assessed whether they favored study results as described in Chan et al. (2004) and others (Mathieu et al., 2009; Rongen & Hannink, 2016; van Lent et al., 2015). We assessed favorableness of discrepancies if the primary outcome(s) of question were completely defined, as only then we were able to make clear assessments on favourableness based on the reported statistics. It was assessed to be *favorable* if a newly reported or upgraded primary outcome was statistically significant or favored study results or a downgraded primary outcome was nonsignificant or did not favor study results. It was assessed to be *unfavorable* if the changed reported primary outcomes were nonsignificant or a registered primary outcome was downgraded and significant. Statistical significance was assessed by

checking whether *p* values and/or 95% confidence intervals indicated $p < .05$ (or, if applicable, on the otherwise specified alpha level).

Registration time point: Trials were considered *prospectively registered* if the registration date preceded the study start date and they were considered *retrospectively registered* if it was registered after the study start date. In cases where the primary outcome was retrospectively changed, trials were considered *prospectively registered with retrospective changes*. We additionally checked whether the changes were justified in the publications. In any case, the latest ("current") version of the primary outcome was used for the study analysis.

Study design: A trial was rated as being a randomized controlled trial (*RCT*) if the study methods reported the trial as an RCT. Otherwise, it was considered not to be a RCT (*no RCT*).

Involvement of pharmaceutical companies: A trial was rated as *reporting the involvement of pharmaceutical companies* if A) one of the authors shows a relationship to a pharmaceutical company either by reporting it in the COI statement, in the acknowledgement section of the preprint or the journal article, or by naming pharmaceutical company as affiliation; or if B) a pharmaceutical company was named as funding source in the registry entry, preprint or journal article; or if C) either in the COI statements, the acknowledgement sections or in the methods section of the publication it was reported that a pharmaceutical company had sponsored the study drug; or if D) a pharmaceutical company was reported to have provided advice (e.g., statistical). All other cases were rated as *not reporting the involvement of pharmaceutical companies*.
