# Supplementary material for: Completeness and consistency of primary outcome reporting in COVID-19 publications in the early pandemic phase: a descriptive study
Source: BMC Med Res Methodol. 2023 Jul 29;23:173. doi: 10.1186/s12874-023-01991-9 (PMC10385884; doi:10.1186/s12874-023-01991-9)
Supplement: Supplementary file 2 — Additional file 2: Table 1. Sample from the open access DIssemination of REgistered COVID-19 Clinical Trials (DIRECCT) database. [file 12874_2023_1991_MOESM2_ESM.pdf]

Additional File 2, Table 1: Sample from the open access DIssemination of REgistered COVID-19 Clinical Trials (DIRECCT) database (https://doi.org/10.5281/zenodo.4669936)

ID	registry ID	preprint reference	journal reference
1	NCT04252885	Y. Li et al., 2020a[1]	Y. Li et al., 2020b[2]
2	ChiCTR2000029853	NA	Ren et al., 2020[3]
3	ChiCTR2000029822	NA	Zhou et al., 2020[4]
4	ChiCTR2000029778	NA	Xin et al., 2020[5]
5	ChiCTR2000029765	D. Wang et al., 2020[6]	NA
6	ChiCTR2000029600	NA	Cai et al., 2020[7]
7	ChiCTR2000029559	Z. Chen et al., 2020c[8]	NA
8	ChiCTR2000029544	Lou et al., 2020[9]	Lou et al., 2021[10]
9	ChiCTR2000029431	Yuan et al., 2020[11]	NA
10	ChiCTR2000029418	Ye, 2020[12]	Ye and G-CHAMPS Collaborative Group, 2020[13]
11	ChiCTR2000030058	NA	j1: M. Wang et al., 2020[14]
		NA	j2: K. Hu et al., 2020[15]
12	ChiCTR2000030054	L. Chen et al., 2020[16]	NA
13	ChiCTR2000030046	NA	Duan et al., 2020[17]
14	ChiCTR2000030007	NA	Cheng et al., 2021[18]
15	ChiCTR2000030001	NA	Wu et al., 2020[19]
16	ChiCTR2000029990	NA	Leng et al., 2020[20]
17	ChiCTR2000029851	Zhong et al., 2020[21]	NA
18	NCT04275245	Bian et al., 2020[22]	NA
19	ChiCTR2000030261	p1: Chu et al., 2020a[23]	NA
		p2: Chu et al., 2020b[24]	NA
20	ChiCTR2000030022	NA	Fan et al., 2021[25]
21	ChiCTR2000030627	S. Chen et al., 2020[26]	NA
22	ChiCTR2000029757	NA	L. Li et al., 2020[27]
23	ChiCTR2000029308	NA	Cao et al., 2020[28]
24	ChiCTR2000030055	Liu et al., 2020a[29]	Liu et al., 2020b[30]
25	ChiCTR2000030939	Zhang et al., 2020[31]	NA
26	ChiCTR2000030254	C. Chen et al., 2020[32]	NA
27	ChiCTR2000029868	Tang et al., 2020a[33]	Tang et al., 2020b[34]
28	ChiCTR2000029496	Zheng et al., 2020[35]	NA
29	EUCTR2020-000890-25-FR	Gautret et al., 2020a[36]	Gautret et al., 2020b[37]

ID	registry ID	preprint reference	journal reference
30	ChiCTR2000030704	F. Hu et al., 2020[38]	NA
31	ChiCTR2000031630	Hong et al., 2020a[39]	Hong et al., 2020b[40]
32	NCT04275414	Pang et al., 2020[41]	NA
33	NCT04320238	Meng et al., 2020[42]	NA
34	NCT04291729	H. Chen et al., 2020a[43]	H. Chen et al., 2020b[44]
35	NCT04310228	NA	Zhao et al., 2021[45]
36	NCT04315480	p1: Sabbatinelli et al., 2020[46]	NA
		p2: Pomponio et al., 2020[47]	NA
37	NCT04317092	Perrone et al., 2020a[48]	Perrone et al., 2020b[49]
38	ChiCTR2000029434	NA	Hu et al., 2021[50]
39	IRCT20100228003449N28	Davoudi-Monfared et al., 2020a[51]	Davoudi-Monfared et al., 2020b[52]
40	IRCT20151227025726N12	NA	Dastan et al., 2020[53]
41	IRCT20190727044343N1	NA	Davoodi et al., 2020[54]
42	NCT04257656	NA	Y. Wang et al., 2020[55]
43	NCT04276688	NA	Hung et al., 2020[56]
44	NCT04358614	NA	Cantini et al., 2020[57]
45	NCT04273763	NA	T. Li et al., 2020[58]
46	NCT04368377	NA	Viecca et al., 2020[59]
47	EUCTR2020-001934-37-ES	Corral-Gudino et al., 2020[60]	NA
48	NCT04324489	NA	Ho et al., 2020[61]
49	NCT04280705	NA	Beigel et al., 2020[62]
50	NCT04326790	NA	Deftereos et al., 2020[63]
51	IRCT20200328046886N1	NA	Abbaspour Kasgari et al., 2020[64]
52	NCT04384380	C.-P. Chen et al., 2020a[65]	C.-P. Chen et al., 2020b[66]
53	NCT04328961	NA	Barnabas et al., 2021[67]
54	NCT04340050	p1: Madariaga et al., 2020b[68]	NA
		p2: Madariaga et al., 2020a[69]	j1: Madariaga et al., 2021[70]
55	NCT04378712	NA	Guan et al., 2020[71]
56	NCT04313127	NA	Zhu et al., 2020[72]
57	NCT04399746	NA	Espitia-Hernandez et al., 2020[73]
58	ChiCTR2000033372	Q. Wang et al., 2020[74]	NA
59	NCT04321421	Perotti et al., 2020a[75]	Perotti et al., 2020b[76]
60	NCT04292730	NA	Spinner et al., 2020[77]
61	NCT04292899	NA	Goldman et al., 2020[78]
62	NCT04322123	NA	Cavalcanti et al., 2020[79]

ID	registry ID	preprint reference	journal reference
63	NCT04329832	NA	Brown et al., 2021[80]
64	NCT04408209	Pappa et al., 2020[81]	NA
65	NCT04408456	NA	Dhibar et al., 2020[82]
66	NCT04343092	Gorial et al., 2020[83]	NA
67	IRCT20200128046294N2	NA	Sadeghi et al., 2020[84]
68	IRCT20200523047550N1	Ghaderkhani et al., 2020[85]	NA
69	JPRN-UMIN000040341	Kageyama et al., 2020a[86]	NA
70	JPRN-UMIN000040407	Kageyama et al., 2020b[87]	NA
71	RPCEC00000313	Venegas-Rodriguez et al., 2020a[88]	Venegas-Rodriguez et al., 2020b[89]
72	RPCEC00000317	Cruz et al., 2020[90]	NA
73	NCT04316377	Lyngbakken et al., 2020a[91]	Lyngbakken et al., 2020b[92]
74	NCT04331795	Strohbehn et al., 2020[93]	NA
75	EUCTR2020-001243-15-BE	Liesenborghs et al., 2020[94]	NA
76	NCT04346446	Bajpai et al., 2020[95]	NA
77	NCT04288102	p1: Shi et al., 2020a[96]	NA
		p2: Shi et al., 2020b[97]	NA
78	NCT04304053	p1: Mitjà et al., 2020c[98]	NA
		p2: Mitjà et al., 2020a[99]	j1: Mitjà et al., 2020b[100]
79	NCT04308668	NA	j1: Boulware et al., 2020[101]
		NA	j2: Skipper et al., 2020[102]
		NA	j3: Lofgren et al., 2020[103]
80	NCT04343729	NA	Jeronimo et al., 2021[104]
81	NCT04346355	NA	Salvarani et al., 2021[105]
82	NCT04349241	Dabbous et al., 2020[106]	NA
83	NCT04356534	AlQahtani et al., 2020[107]	NA
84	NCT04356937	Stone et al., 2020[108]	NA
85	NCT04369742	NA	Ulrich et al., 2020[109]
86	NCT04441424	Rasheed et al., 2020a[110]	Rasheed et al., 2020b[111]
87	NCT04381936	p1: P. Horby et al., 2020[112]	j1: RECOVERY Collaborative Group, 2021[113]
		p2: P. W. Horby et al., 2020[114]	NA
		NA	j2: RECOVERY Collaborative Group, 2020a[115]
		NA	j3: RECOVERY Collaborative Group, 2020b[116]

Note. p# = sequential number of preprint per trial; j# = sequential number of journal per trial.

1. Li Y, Xie Z, Lin W, et al. *An Exploratory Randomized Controlled Study on the Efficacy and Safety of Lopinavir/Ritonavir or Arbidol Treating Adult Patients Hospitalized with Mild/Moderate COVID-19 (ELACOI)*. Infectious Diseases (except HIV/AIDS); 2020. doi:10.1101/2020.03.19.20038984
2. Li Y, Xie Z, Lin W, et al. Efficacy and Safety of Lopinavir/Ritonavir or Arbidol in Adult Patients with Mild/Moderate COVID-19: An Exploratory Randomized Controlled Trial. *Med*. 2020;1(1):105-113.e4. doi:10.1016/j.medj.2020.04.001
3. Ren Z, Luo H, Yu Z, et al. A Randomized, Open‐Label, Controlled Clinical Trial of Azvudine Tablets in the Treatment of Mild and Common COVID‐19, a Pilot Study. *Adv Sci*. 2020;7(19):2001435. doi:10.1002/advs.202001435
4. Zhou LK, Zhou Z, Jiang XM, et al. Absorbed plant MIR2911 in honeysuckle decoction inhibits SARS-CoV-2 replication and accelerates the negative conversion of infected patients. *Cell Discov*. 2020;6(1):54. doi:10.1038/s41421-020-00197-3
5. Xin S, Cheng X, Zhu B, et al. Clinical retrospective study on the efficacy of Qingfei Paidu decoction combined with Western medicine for COVID-19 treatment. *Biomedicine & Pharmacotherapy*. 2020;129:110500. doi:10.1016/j.biopha.2020.110500
6. Wang D, Fu B, Peng Z, et al. Tocilizumab Ameliorates the Hypoxia in COVID-19 Moderate Patients with Bilateral Pulmonary Lesions: A Randomized, Controlled, Open-Label, Multicenter Trial. *SSRN Journal*. Published online 2020. doi:10.2139/ssrn.3667681
7. Cai Q, Yang M, Liu D, et al. Experimental Treatment with Favipiravir for COVID-19: An Open-Label Control Study. *Engineering*. 2020;6(10):1192-1198. doi:10.1016/j.eng.2020.03.007
8. Chen Z, Hu J, Zhang Z, et al. *Efficacy of Hydroxychloroquine in Patients with COVID-19: Results of a Randomized Clinical Trial*. Epidemiology; 2020. doi:10.1101/2020.03.22.20040758
9. Lou Y, Liu L, Yao H, et al. *Clinical Outcomes and Plasma Concentrations of Baloxavir Marboxil and Favipiravir in COVID-19 Patients: An Exploratory Randomized, Controlled Trial*. Pharmacology and Therapeutics; 2020. doi:10.1101/2020.04.29.20085761
10. Lou Y, Liu L, Yao H, et al. Clinical Outcomes and Plasma Concentrations of Baloxavir Marboxil and Favipiravir in COVID-19 Patients: An Exploratory Randomized, Controlled Trial. *European Journal of Pharmaceutical Sciences*. 2021;157:105631. doi:10.1016/j.ejps.2020.105631
11. Yuan X, Yi W, Liu B, et al. *Pulmonary Radiological Change of COVID-19 Patients with ^{99m} Tc-MDP Treatment*. Public and Global Health; 2020. doi:10.1101/2020.04.07.20054767
12. Ye Y an. *Guideline-Based Chinese Herbal Medicine Treatment plus Standard Care for Severe Coronavirus Disease 2019 (G-CHAMPS): Evidence from China*. Intensive Care and Critical Care Medicine; 2020. doi:10.1101/2020.03.27.20044974
13. Ye Y an, G-CHAMPS Collaborative Group. Guideline-Based Chinese Herbal Medicine Treatment Plus Standard Care for Severe Coronavirus Disease 2019 (G-CHAMPS): Evidence From China. *Front Med*. 2020;7:256. doi:10.3389/fmed.2020.00256
14. Wang M, Zhao Y, Hu W, et al. Treatment of Coronavirus Disease 2019 Patients With Prolonged Postsymptomatic Viral Shedding With Leflunomide: A Single-center Randomized Controlled Clinical Trial. *Clinical Infectious Diseases*. Published online September 21, 2020:ciaa1417. doi:10.1093/cid/ciaa1417
15. Hu K, Wang M, Zhao Y, et al. A Small-Scale Medication of Leflunomide as a Treatment of COVID-19 in an Open-Label Blank-Controlled Clinical Trial. *Virol Sin*. 2020;35(6):725-733. doi:10.1007/s12250-020-00258-7
16. Chen L, Zhang ZY, Fu JG, et al. *Efficacy and Safety of Chloroquine or Hydroxychloroquine in Moderate Type of COVID-19: A Prospective Open-Label Randomized Controlled Study*. Infectious Diseases (except HIV/AIDS); 2020.

doi:10.1101/2020.06.19.20136093
17. Duan K, Liu B, Li C, et al. Effectiveness of convalescent plasma therapy in severe COVID-19 patients. *Proc Natl Acad Sci USA*. 2020;117(17):9490-9496. doi:10.1073/pnas.2004168117
18. Cheng L ling, Guan W jie, Duan C yang, et al. Effect of Recombinant Human Granulocyte Colony–Stimulating Factor for Patients With Coronavirus Disease 2019 (COVID-19) and Lymphopenia: A Randomized Clinical Trial. *JAMA Intern Med*. 2021;181(1):71. doi:10.1001/jamainternmed.2020.5503
19. Wu X, Yu K, Wang Y, et al. Efficacy and Safety of Triazavirin Therapy for Coronavirus Disease 2019: A Pilot Randomized Controlled Trial. *Engineering*. 2020;6(10):1185-1191. doi:10.1016/j.eng.2020.08.011
20. Leng Z, Zhu R, Hou W, et al. Transplantation of ACE2- Mesenchymal Stem Cells Improves the Outcome of Patients with COVID-19 Pneumonia. *Aging and disease*. 2020;11(2):216. doi:10.14336/AD.2020.0228
21. Zhong M, Sun A, Xiao T, et al. *A Randomized, Single-Blind, Group Sequential, Active-Controlled Study to Evaluate the Clinical Efficacy and Safety of α-Lipoic Acid for Critically Ill Patients with Coronavirus Disease 2019(COVID-19)*. Pharmacology and Therapeutics; 2020. doi:10.1101/2020.04.15.20066266
22. Bian H, Zheng ZH, Wei D, et al. *Meplazumab Treats COVID-19 Pneumonia: An Open-Labelled, Concurrent Controlled Add-on Clinical Trial*. Infectious Diseases (except HIV/AIDS); 2020. doi:10.1101/2020.03.21.20040691
23. Chu M, Wang H, Bian L, et al. Nebulization Therapy for COVID-19 Pneumonia with Embryonic Mesenchymal Stem Cells-Derived Exosomes. *SSRN Journal*. Published online 2020. doi:10.2139/ssrn.3678558
24. Chu M, Wang H, Bian L, et al. *Nebulization Therapy for COVID-19 Pneumonia with Embryonic Mesenchymal Stem Cells-Derived Exosomes*. In Review; 2020. doi:10.21203/rs.3.rs-99753/v1
25. Fan S, Zhen Q, Chen C, et al. Clinical efficacy of low-dose emetine for patients with COVID-19: a real-world study. *Journal of Bio-X Research*. 2021;4(2):53-59. doi:10.1097/JBR.0000000000000076
26. Chen S, Lu C, Li P, et al. *Effectiveness of Convalescent Plasma for Treatment of COVID-19 Patients*. Respiratory Medicine; 2020. doi:10.1101/2020.08.02.20166710
27. Li L, Zhang W, Hu Y, et al. Effect of Convalescent Plasma Therapy on Time to Clinical Improvement in Patients With Severe and Life-threatening COVID-19: A Randomized Clinical Trial. *JAMA*. 2020;324(5):460. doi:10.1001/jama.2020.10044
28. Cao B, Wang Y, Wen D, et al. A Trial of Lopinavir–Ritonavir in Adults Hospitalized with Severe Covid-19. *N Engl J Med*. 2020;382(19):1787-1799. doi:10.1056/NEJMoa2001282
29. Liu X, Li Z, Liu S, et al. *Therapeutic Effects of Dipyridamole on COVID-19 Patients with Coagulation Dysfunction*. Infectious Diseases (except HIV/AIDS); 2020. doi:10.1101/2020.02.27.20027557
30. Liu X, Li Z, Liu S, et al. Potential therapeutic effects of dipyridamole in the severely ill patients with COVID-19. *Acta Pharmaceutica Sinica B*. 2020;10(7):1205-1215. doi:10.1016/j.apsb.2020.04.008
31. Zhang H, Zhao Y, Jiang X, et al. *Preliminary Evaluation of the Safety and Efficacy of Oral Human Antimicrobial Peptide LL-37 in the Treatment of Patients of COVID-19, a Small-Scale, Single-Arm, Exploratory Safety Study*. Infectious Diseases (except HIV/AIDS); 2020. doi:10.1101/2020.05.11.20064584
32. Chen C, Zhang Y, Huang J, et al. *Favipiravir versus Arbidol for COVID-19: A Randomized Clinical Trial*. Infectious Diseases (except HIV/AIDS); 2020. doi:10.1101/2020.03.17.20037432
33. Tang W, Cao Z, Han M, et al. *Hydroxychloroquine in Patients Mainly with Mild to*

Moderate COVID–19: An Open–Label, Randomized, Controlled Trial. Public and Global Health; 2020. doi:10.1101/2020.04.10.20060558
34. Tang W, Cao Z, Han M, et al. Hydroxychloroquine in patients with mainly mild to moderate coronavirus disease 2019: open label, randomised controlled trial. *BMJ*. Published online May 14, 2020:m1849. doi:10.1136/bmj.m1849
35. Zheng F, Zhou Y, Zhou Z, et al. *A Novel Protein Drug, Novaferon, as the Potential Antiviral Drug for COVID-19*. Infectious Diseases (except HIV/AIDS); 2020. doi:10.1101/2020.04.24.20077735
36. Gautret P, Lagier JC, Parola P, et al. *Hydroxychloroquine and Azithromycin as a Treatment of COVID-19: Results of an Open-Label Non-Randomized Clinical Trial*. Infectious Diseases (except HIV/AIDS); 2020. doi:10.1101/2020.03.16.20037135
37. Gautret P, Lagier JC, Parola P, et al. Hydroxychloroquine and azithromycin as a treatment of COVID-19: results of an open-label non-randomized clinical trial. *International Journal of Antimicrobial Agents*. 2020;56(1):105949. doi:10.1016/j.ijantimicag.2020.105949
38. Hu F, Chen J, Chen H, et al. *Chansu Injection Improves the Respiratory Function of Severe COVID-19 Patients*. Infectious Diseases (except HIV/AIDS); 2020. doi:10.1101/2020.05.20.20107607
39. Hong W, Chen Y, You K, et al. *Celebrex Adjuvant Therapy on COVID-19: An Experimental Study*. Public and Global Health; 2020. doi:10.1101/2020.05.05.20077610
40. Hong W, Chen Y, You K, et al. Celebrex Adjuvant Therapy on Coronavirus Disease 2019: An Experimental Study. *Front Pharmacol*. 2020;11:561674. doi:10.3389/fphar.2020.561674
41. Pang J, Xu F, Aondio G, et al. *Efficacy and Tolerability of Bevacizumab in Patients with Severe Covid -19*. Infectious Diseases (except HIV/AIDS); 2020. doi:10.1101/2020.07.26.20159756
42. Meng Z, Wang T, Chen L, et al. *An Experimental Trial of Recombinant Human Interferon Alpha Nasal Drops to Prevent COVID-19 in Medical Staff in an Epidemic Area*. Infectious Diseases (except HIV/AIDS); 2020. doi:10.1101/2020.04.11.20061473
43. Chen H, Zhang Z, Wang L, et al. *First Clinical Study Using HCV Protease Inhibitor Danoprevir to Treat Naïve and Experienced COVID-19 Patients*. Infectious Diseases (except HIV/AIDS); 2020. doi:10.1101/2020.03.22.20034041
44. Chen H, Zhang Z, Wang L, et al. First clinical study using HCV protease inhibitor danoprevir to treat COVID-19 patients. *Medicine*. 2020;99(48):e23357. doi:10.1097/MD.0000000000023357
45. Zhao H, Zhu Q, Zhang C, et al. Tocilizumab combined with favipiravir in the treatment of COVID-19: A multicenter trial in a small sample size. *Biomedicine & Pharmacotherapy*. 2021;133:110825. doi:10.1016/j.biopha.2020.110825
46. Sabbatinelli J, Giuliani A, Matacchione G, et al. *Decreased Serum Levels of Inflammaging Marker MiR-146a Are Associated with Clinical Response to Tocilizumab in COVID-19 Patients*. Infectious Diseases (except HIV/AIDS); 2020. doi:10.1101/2020.07.11.20151365
47. Pomponio G, Ferrarini A, Bonifazi M, et al. *Tocilizumab in Covid-19 Interstitial Pneumonia: A Phase II Pilot Study*. In Review; 2020. doi:10.21203/rs.3.rs-42117/v1
48. Perrone F, Piccirillo MC, Ascierto PA, et al. *Tocilizumab for Patients with COVID-19 Pneumonia. The TOCIVID-19 Prospective Phase 2 Trial*. Infectious Diseases (except HIV/AIDS); 2020. doi:10.1101/2020.06.01.20119149
49. Perrone F, Piccirillo MC, Ascierto PA, et al. Tocilizumab for patients with COVID-19 pneumonia. The single-arm TOCIVID-19 prospective trial. *J Transl Med*. 2020;18(1):405. doi:10.1186/s12967-020-02573-9
50. Hu K, Guan W jie, Bi Y, et al. Efficacy and safety of Lianhuaqingwen capsules, a repurposed Chinese herb, in patients with coronavirus disease 2019: A multicenter,

prospective, randomized controlled trial. *Phytomedicine*. 2021;85:153242. doi:10.1016/j.phymed.2020.153242
51. Davoudi-Monfared E, Rahmani H, Khalili H, et al. *Efficacy and Safety of Interferon Beta-1a in Treatment of Severe COVID-19: A Randomized Clinical Trial*. Infectious Diseases (except HIV/AIDS); 2020. doi:10.1101/2020.05.28.20116467
52. Davoudi-Monfared E, Rahmani H, Khalili H, et al. A Randomized Clinical Trial of the Efficacy and Safety of Interferon β-1a in Treatment of Severe COVID-19. *Antimicrob Agents Chemother*. 2020;64(9). doi:10.1128/AAC.01061-20
53. Dastan F, Nadji SA, Saffaei A, et al. Subcutaneous administration of interferon beta-1a for COVID-19: A non-controlled prospective trial. *International Immunopharmacology*. 2020;85:106688. doi:10.1016/j.intimp.2020.106688
54. Davoodi L, Abedi SM, Salehifar E, et al. Febuxostat therapy in outpatients with suspected COVID‐19: A clinical trial. *Int J Clin Pract*. 2020;74(11). doi:10.1111/ijcp.13600
55. Wang Y, Zhang D, Du G, et al. Remdesivir in adults with severe COVID-19: a randomised, double-blind, placebo-controlled, multicentre trial. *The Lancet*. 2020;395(10236):1569-1578. doi:10.1016/S0140-6736(20)31022-9
56. Hung IFN, Lung KC, Tso EYK, et al. Triple combination of interferon beta-1b, lopinavir–ritonavir, and ribavirin in the treatment of patients admitted to hospital with COVID-19: an open-label, randomised, phase 2 trial. *The Lancet*. 2020;395(10238):1695-1704. doi:10.1016/S0140-6736(20)31042-4
57. Cantini F, Niccoli L, Matarrese D, Nicastri E, Stobbione P, Goletti D. Baricitinib therapy in COVID-19: A pilot study on safety and clinical impact. *Journal of Infection*. 2020;81(2):318-356. doi:10.1016/j.jinf.2020.04.017
58. Li T, Sun L, Zhang W, et al. Bromhexine Hydrochloride Tablets for the Treatment of Moderate COVID‐19: An Open‐Label Randomized Controlled Pilot Study. *Clin Transl Sci*. 2020;13(6):1096-1102. doi:10.1111/cts.12881
59. Viecca M, Radovanovic D, Forleo GB, Santus P. Enhanced platelet inhibition treatment improves hypoxemia in patients with severe Covid-19 and hypercoagulability. A case control, proof of concept study. *Pharmacological Research*. 2020;158:104950. doi:10.1016/j.phrs.2020.104950
60. Corral-Gudino L, Bahamonde A, Arnaiz-Revillas F, et al. *GLUCOCOVID: A Controlled Trial of Methylprednisolone in Adults Hospitalized with COVID-19 Pneumonia*. Infectious Diseases (except HIV/AIDS); 2020. doi:10.1101/2020.06.17.20133579
61. Ho JHC, Zhao Y, Liu Z, et al. Resolution of Coronavirus Disease 2019 Infection and Pulmonary Pathology With Nebulized DAS181: A Pilot Study. *Critical Care Explorations*. 2020;2(10):e0263. doi:10.1097/CCE.0000000000000263
62. Beigel JH, Tomashek KM, Dodd LE, et al. Remdesivir for the Treatment of Covid-19 — Final Report. *N Engl J Med*. 2020;383(19):1813-1826. doi:10.1056/NEJMoa2007764
63. Deftereos SG, Giannopoulos G, Vrachatis DA, et al. Effect of Colchicine vs Standard Care on Cardiac and Inflammatory Biomarkers and Clinical Outcomes in Patients Hospitalized With Coronavirus Disease 2019: The GRECCO-19 Randomized Clinical Trial. *JAMA Netw Open*. 2020;3(6):e2013136. doi:10.1001/jamanetworkopen.2020.13136
64. Abbaspour Kasgari H, Moradi S, Shabani AM, et al. Evaluation of the efficacy of sofosbuvir plus daclatasvir in combination with ribavirin for hospitalized COVID-19 patients with moderate disease compared with standard care: a single-centre, randomized controlled trial. *Journal of Antimicrobial Chemotherapy*. 2020;75(11):3373-3378. doi:10.1093/jac/dkaa332
65. Chen CP, Lin YC, Chen TC, et al. *A Multicenter, Randomized, Open-Label, Controlled Trial to Evaluate the Efficacy and Tolerability of Hydroxychloroquine and a Retrospective Study in Adult Patients with Mild to Moderate Coronavirus Disease 2019 (COVID-19)*. Infectious Diseases (except HIV/AIDS); 2020. doi:10.1101/2020.07.08.20148841

66. Chen CP, Lin YC, Chen TC, et al. A multicenter, randomized, open-label, controlled trial to evaluate the efficacy and tolerability of hydroxychloroquine and a retrospective study in adult patients with mild to moderate coronavirus disease 2019 (COVID-19). Atkin SL, ed. *PLoS ONE*. 2020;15(12):e0242763. doi:10.1371/journal.pone.0242763
67. Barnabas RV, Brown ER, Bershteyn A, et al. Hydroxychloroquine as Postexposure Prophylaxis to Prevent Severe Acute Respiratory Syndrome Coronavirus 2 Infection: A Randomized Trial. *Ann Intern Med*. 2021;174(3):344-352. doi:10.7326/M20-6519
68. Madariaga ML, Schrantz S, Jansen MO, et al. Integrated COVID-19 Convalescent Plasma Treatment and Antibody Research Program at a Single Academic Medical Center. *SSRN Journal*. Published online 2020. doi:10.2139/ssrn.3605131
69. Madariaga ML, Guthmiller JJ, Schrantz S, et al. *Clinical Predictors of Donor Antibody Titer and Correlation with Recipient Antibody Response in a COVID-19 Convalescent Plasma Clinical Trial*. Infectious Diseases (except HIV/AIDS); 2020. doi:10.1101/2020.06.21.20132944
70. Madariaga MLL, Guthmiller JJ, Schrantz S, et al. Clinical predictors of donor antibody titre and correlation with recipient antibody response in a COVID‐19 convalescent plasma clinical trial. *J Intern Med*. 2021;289(4):559-573. doi:10.1111/joim.13185
71. Guan WJ, Wei CH, Chen AL, et al. Hydrogen/oxygen mixed gas inhalation improves disease severity and dyspnea in patients with Coronavirus disease 2019 in a recent multicenter, open-label clinical trial. *J Thorac Dis*. 2020;12(6):3448-3452. doi:10.21037/jtd-2020-057
72. Zhu FC, Li YH, Guan XH, et al. Safety, tolerability, and immunogenicity of a recombinant adenovirus type-5 vectored COVID-19 vaccine: a dose-escalation, open-label, non-randomised, first-in-human trial. *The Lancet*. 2020;395(10240):1845-1854. doi:10.1016/S0140-6736(20)31208-3
73. Espitia-Hernandez G, Munguia L, Diaz-Chiguer D, Lopez-Elizalde R, Jimenez-Ponce F. Effects of Ivermectin-azithromycin-cholecalciferol combined therapy on COVID-19 infected patients: A proof of concept study. Published online 2020. Accessed November 17, 2021. https://www.biomedres.info/abstract/effects-of-ivermectinazithromycincholecalciferol-combined-therapy-on-covid19-infected-patients-a-proof-of-concept-study-14435.html
74. Wang Q, Guo H, Li Y, et al. *Efficacy and Safety of Leflunomide for Refractory COVID-19: An Open-Label Controlled Study*. Infectious Diseases (except HIV/AIDS); 2020. doi:10.1101/2020.05.29.20114223
75. Perotti C, Baldanti F, Bruno R, et al. *Mortality Reduction in 46 Severe Covid-19 Patients Treated with Hyperimmune Plasma. A Proof of Concept Single Arm Multicenter Interventional Trial*. Infectious Diseases (except HIV/AIDS); 2020. doi:10.1101/2020.05.26.20113373
76. Perotti C, Baldanti F, Bruno R, et al. Mortality reduction in 46 severe Covid-19 patients treated with hyperimmune plasma. A proof of concept single arm multicenter trial. *Haematologica*. 2020;105(12):2834-2840. doi:10.3324/haematol.2020.261784
77. Spinner CD, Gottlieb RL, Criner GJ, et al. Effect of Remdesivir vs Standard Care on Clinical Status at 11 Days in Patients With Moderate COVID-19: A Randomized Clinical Trial. *JAMA*. 2020;324(11):1048. doi:10.1001/jama.2020.16349
78. Goldman JD, Lye DCB, Hui DS, et al. Remdesivir for 5 or 10 Days in Patients with Severe Covid-19. *N Engl J Med*. 2020;383(19):1827-1837. doi:10.1056/NEJMoa2015301
79. Cavalcanti AB, Zampieri FG, Rosa RG, et al. Hydroxychloroquine with or without Azithromycin in Mild-to-Moderate Covid-19. *N Engl J Med*. 2020;383(21):2041-2052. doi:10.1056/NEJMoa2019014
80. Brown SM, Peltan I, Kumar N, et al. Hydroxychloroquine versus Azithromycin for Hospitalized Patients with COVID-19. Results of a Randomized, Active Comparator Trial.

Annals ATS. 2021;18(4):590-597. doi:10.1513/AnnalsATS.202008-940OC
81. Pappa V, Politou M, Papageorgiou SG, et al. *A Prospective Phase II Study on The Use Of Convalescent Plasma Monotherapy For The Treatment of Severe Covid-19 Disease: A Preliminary Report.* In Review; 2020. doi:10.21203/rs.3.rs-64465/v1
82. Dhibar DP, Arora N, Kakkar A, et al. Post-exposure prophylaxis with hydroxychloroquine for the prevention of COVID-19, a myth or a reality? The PEP-CQ Study. *International Journal of Antimicrobial Agents*. 2020;56(6):106224. doi:10.1016/j.ijantimicag.2020.106224
83. Gorial FI, Mashhadani S, Sayaly HM, et al. *Effectiveness of Ivermectin as Add-on Therapy in COVID-19 Management (Pilot Trial)*. Infectious Diseases (except HIV/AIDS); 2020. doi:10.1101/2020.07.07.20145979
84. Sadeghi A, Ali Asgari A, Norouzi A, et al. Sofosbuvir and daclatasvir compared with standard of care in the treatment of patients admitted to hospital with moderate or severe coronavirus infection (COVID-19): a randomized controlled trial. *Journal of Antimicrobial Chemotherapy*. 2020;75(11):3379-3385. doi:10.1093/jac/dkaa334
85. Ghaderkhani S, Khaneshan A salami, Salami A, et al. *Efficacy and Safety of Arbidol in Treatment of Patients with COVID-19 Infection: A Randomized Clinical Trial*. In Review; 2020. doi:10.21203/rs.3.rs-91430/v1
86. Kageyama Y, Aida K, Kawauchi K, et al. *Qing Fei Pai Du Tang, a Chinese Multi-Herbal Medicine Formulated against COVID-19, Elevates the Plasma Levels of IL-1β, IL-18, TNF-α, and IL-8*. Infectious Diseases (except HIV/AIDS); 2020. doi:10.1101/2020.07.13.20146175
87. Kageyama Y, Aida K, Kawauchi K, et al. *Jinhua Qinggan Granule, a Chinese Herbal Medicine against COVID-19, Induces Rapid Changes in the Plasma Levels of IL-6 and IFN-γ*. Infectious Diseases (except HIV/AIDS); 2020. doi:10.1101/2020.06.08.20124453
88. Venegas-Rodriguez R, Santana-Sanchez R, Peña-Ruiz R, et al. *CIGB-258 Immunomodulatory Peptide: A Novel Promising Treatment for Critical and Severe COVID-19 Patients*. Infectious Diseases (except HIV/AIDS); 2020. doi:10.1101/2020.05.27.20110601
89. Venegas-Rodriguez R, Santana-Sanchez R, Peña-Ruiz R, et al. CIGB-258 Immunomodulatory Peptide: Compassionate Use for Critical and Severe COVID-19 Patients. *Austin J Pharmacol Ther*. 2020;8(1):1119.
90. Cruz LR, Baladrón I, Rittoles A, et al. *Treatment with an Anti-CK2 Synthetic Peptide Improves Clinical Response in Covid-19 Patients with Pneumonia. A Randomized and Controlled Clinical Trial*. Pharmacology and Therapeutics; 2020. doi:10.1101/2020.09.03.20187112
91. Lyngbakken MN, Berdal JE, Eskesen A, et al. *A Pragmatic Randomized Controlled Trial Reports the Efficacy of Hydroxychloroquine on Coronavirus Disease 2019 Viral Kinetics*. In Review; 2020. doi:10.21203/rs.3.rs-44055/v1
92. Lyngbakken MN, Berdal JE, Eskesen A, et al. A pragmatic randomized controlled trial reports lack of efficacy of hydroxychloroquine on coronavirus disease 2019 viral kinetics. *Nat Commun*. 2020;11(1):5284. doi:10.1038/s41467-020-19056-6
93. Strohbehn GW, Heiss BL, Rouhani SJ, et al. *COVIDOSE: Low-Dose Tocilizumab in the Treatment of Covid-19*. Infectious Diseases (except HIV/AIDS); 2020. doi:10.1101/2020.07.20.20157503
94. Liesenborghs L, Spriet I, Jochmans D, et al. Itraconazole for COVID-19: Preclinical Studies and a Proof-of-Concept Pilot Clinical Study. *SSRN Journal*. Published online 2020. doi:10.2139/ssrn.3731461
95. Bajpai M, Kumar S, Maheshwari A, et al. *Efficacy of Convalescent Plasma Therapy Compared to Fresh Frozen Plasma in Severely Ill COVID-19 Patients: A Pilot Randomized Controlled Trial*. Infectious Diseases (except HIV/AIDS); 2020.

doi:10.1101/2020.10.25.20219337
96. Shi L, Huang H, Lu X, et al. *Treatment with Human Umbilical Cord-Derived Mesenchymal Stem Cells for COVID-19 Patients with Lung Damage: A Randomised, Double-Blind, Placebo-Controlled Phase 2 Trial*. Infectious Diseases (except HIV/AIDS); 2020. doi:10.1101/2020.10.15.20213553
97. Shi L, Huang H, Lu X, et al. Treatment with Human Umbilical Cord-Derived Mesenchymal Stem Cells for Severe COVID-19 Patients with Lung Damage: A Randomised, Double-Blind, Placebo-Controlled Phase 2 Trial. *SSRN Journal*. Published online 2020. doi:10.2139/ssrn.3680611
98. Mitjà O, Ubals M, Corbacho-Monné M, et al. *A Cluster-Randomized Trial of Hydroxychloroquine as Prevention of Covid-19 Transmission and Disease*. Epidemiology; 2020. doi:10.1101/2020.07.20.20157651
99. Mitjà O, Corbacho M, G-Beiras C, et al. Hydroxychloroquine Alone or in Combination with Cobicistat-Boosted Darunavir for Treatment of Mild COVID-19: A Cluster-Randomized Clinical Trial. *SSRN Journal*. Published online 2020. doi:10.2139/ssrn.3615997
100. Mitjà O, Corbacho-Monné M, Ubals M, et al. Hydroxychloroquine for Early Treatment of Adults With Mild Coronavirus Disease 2019: A Randomized, Controlled Trial. *Clinical Infectious Diseases*. Published online July 16, 2020:ciaa1009. doi:10.1093/cid/ciaa1009
101. Boulware DR, Pullen MF, Bangdiwala AS, et al. A Randomized Trial of Hydroxychloroquine as Postexposure Prophylaxis for Covid-19. *N Engl J Med*. 2020;383(6):517-525. doi:10.1056/NEJMoa2016638
102. Skipper CP, Pastick KA, Engen NW, et al. Hydroxychloroquine in Nonhospitalized Adults With Early COVID-19: A Randomized Trial. *Annals of Internal Medicine*. 2020;173(8):623-631. doi:10.7326/M20-4207
103. Lofgren SM, Nicol MR, Bangdiwala AS, et al. Safety of Hydroxychloroquine Among Outpatient Clinical Trial Participants for COVID-19. *Open Forum Infectious Diseases*. 2020;7(11):ofaa500. doi:10.1093/ofid/ofaa500
104. Jeronimo CMP, Farias MEL, Val FFA, et al. Methylprednisolone as Adjunctive Therapy for Patients Hospitalized With Coronavirus Disease 2019 (COVID-19; Metcovid): A Randomized, Double-blind, Phase IIb, Placebo-controlled Trial. *Clinical Infectious Diseases*. 2021;72(9):e373-e381. doi:10.1093/cid/ciaa1177
105. Salvarani C, Dolci G, Massari M, et al. Effect of Tocilizumab vs Standard Care on Clinical Worsening in Patients Hospitalized With COVID-19 Pneumonia: A Randomized Clinical Trial. *JAMA Intern Med*. 2021;181(1):24. doi:10.1001/jamainternmed.2020.6615
106. Dabbous HM, El-Sayed MH, Assal GE, et al. *A Randomized Controlled Study Of Favipiravir Vs Hydroxychloroquine In COVID-19 Management: What Have We Learned So Far?* In Review; 2020. doi:10.21203/rs.3.rs-83677/v1
107. AlQahtani M, Abdulrahman A, Almadani A, et al. *Randomized Controlled Trial of Convalescent Plasma Therapy against Standard Therapy in Patients with Severe COVID-19 Disease*. Infectious Diseases (except HIV/AIDS); 2020. doi:10.1101/2020.11.02.20224303
108. Stone JH, Frigault MJ, Serling-Boyd NJ, et al. Efficacy of Tocilizumab in Patients Hospitalized with Covid-19. *N Engl J Med*. 2020;383(24):2333-2344. doi:10.1056/NEJMoa2028836
109. Ulrich RJ, Troxel AB, Carmody E, et al. Treating COVID-19 With Hydroxychloroquine (TEACH): A Multicenter, Double-Blind Randomized Controlled Trial in Hospitalized Patients. *Open Forum Infectious Diseases*. 2020;7(10):ofaa446. doi:10.1093/ofid/ofaa446
110. Rasheed AM, Fatak DF, Hashim HA, et al. *The Therapeutic Effectiveness of Convalescent Plasma Therapy on Treating COVID-19 Patients Residing in Respiratory Care Units in Baghdad, Iraq*. Infectious Diseases (except HIV/AIDS); 2020. doi:10.1101/2020.06.24.20121905

111. Rasheed AM, Fatak DF, Hashim HA, et al. The therapeutic potential of convalescent plasma therapy on treating critically-ill COVID-19 patients residing in respiratory care units in hospitals in Baghdad, Iraq. *Infez Med*. 2020;28(3):357-366.
112. Horby P, Lim WS, Emberson J, et al. *Effect of Dexamethasone in Hospitalized Patients with COVID-19 – Preliminary Report*. Infectious Diseases (except HIV/AIDS); 2020. doi:10.1101/2020.06.22.20137273
113. RECOVERY Collaborative Group. Dexamethasone in Hospitalized Patients with Covid-19. *N Engl J Med*. 2021;384(8):693-704. doi:10.1056/NEJMoa2021436
114. Horby PW, Roddick A, Spata E, et al. *Azithromycin in Hospitalised Patients with COVID-19 (RECOVERY): A Randomised, Controlled, Open-Label, Platform Trial*. Infectious Diseases (except HIV/AIDS); 2020. doi:10.1101/2020.12.10.20245944
115. RECOVERY Collaborative Group. Lopinavir-ritonavir in patients admitted to hospital with COVID-19 (RECOVERY): a randomised, controlled, open-label, platform trial. *Lancet*. 2020;396(10259):1345-1352. doi:10.1016/S0140-6736(20)32013-4
116. RECOVERY Collaborative Group. Effect of Hydroxychloroquine in Hospitalized Patients with Covid-19. *N Engl J Med*. 2020;383(21):2030-2040. doi:10.1056/NEJMoa2022926
