# Supplementary material for: Completeness and consistency of primary outcome reporting in COVID-19 publications in the early pandemic phase: a descriptive study
Source: BMC Med Res Methodol. 2023 Jul 29;23:173. doi: 10.1186/s12874-023-01991-9 (PMC10385884; doi:10.1186/s12874-023-01991-9)
Supplement: Supplementary file 3 — Additional file 3: Table 2. Primary outcome definitions in registry, preprint and journal articles and rating of completeness and discrepancies in the 87 included trials. [file 12874_2023_1991_MOESM3_ESM.pdf]

Additional File 3, Table 2. Primary outcome definitions in registry, preprint and journal articles and rating of completeness and discrepancies in the 87 included trials.

ID		Registry entry (R)	C	p#PO#	Preprint (P)	C	j#PO#	Journal Article (J)	C	Discrepancy rating	
										R vs. P	R vs. J
1	rPO1	The rate of virus inhibition [Time Frame: Day 0, 2, 4, 7, 10, 14 and 21] Novel coronaviral nucleic acid is measured in nose / throat swab at each time point	ic	NA	*[The secondary outcomes included 1) the rate of positive-to-negative conversion of SARS-CoV-2 nucleic acid at day 7 of treatment; 2) the rate of positive-to-negative conversion of SARS-CoV-2 nucleic acid at day 14 [...]]*		j1PO1	The primary outcome was the rate of positive-to-negative conversion of SARS-CoV-2nucleic acid from the initiation of treatment to day 21, with the enrollment day as the first day of treatment. [...] SARS-CoV-2 nucleic acid was detected by real-time fluorescence reverse transcriptional polymerase chain reaction (RT-PCR)	c	NA	NA
	NA	NA		p1PO1	The primary outcome was the time of positive-to-negative conversion of SARS-CoV-2 nucleic acid from the initiation of treatment to day 21, with the enrollment day as the first day of treatment. [...] SARS-CoV-2 nucleic acid was detected by real-time fluorescence reverse transcriptional polymerase chain reaction (RT-PCR)	c	NA	NA		NA	NA
2	rPO1	time and rate of temperature return to normal	ic	NA	NA			*[Secondary outcomes were [...] the time required for the body temper-ature returning to normal [...].]*		NA	NA
	rPO2	time and rate of improvement of respiratory symptoms and signs (lung rhones, cough, sputum, sore throat, etc.)	ic	NA	NA			*[Secondary outcomes were[...] the improvement in respiratory symptoms and signs.]*		NA	NA
	rPO3	time and rate of improvement of diarrhea, myalgia, fatigue and other symptoms	ic	NA	NA			NA		NA	NA
	rPO4	time and rate of pulmonary imaging improvement	ic	NA	NA			*[Secondary outcomes were the im-provement rate of chest CT images [...] The improvement of chest CT images was defined as a signif-icant reduction in the range of lesions and inflammation. The results for*		NA	NA

ID		Registry entry (R)	C	p#PO#	Preprint (P)	C	j#PO#	Journal Article (J)	C	Discrepancy rating	
										R vs. P	R vs. J
								this outcome were double-checked by two radiologists]			
	rPO5	time and rate of change to negative COVID-19 nucleic acid test	ic	NA	NA		j1PO1	The primary outcomes were the rate of nucleic acid negativity conversion of SARS-CoV-19 and the negativity conversion time. Nucleic acid conversion rate was defined as the ratio of patients with negativity nucleic acid testing in FNC group to all patients in FNC group at a certain point in the follow-up process.	ic	NA	NA
	rPO6	time and rate of improvement of oxygenation measurement	ic	NA	NA			NA		NA	NA
	rPO7	improvement time and rate of CD4 count	ic	NA	NA			NA		NA	NA
	rPO8	rate of mild/modorate type to severe type, rate of severe type to critical type	ic	NA	NA			NA		NA	NA
	rPO9	length of hospitalization	ic	NA	NA			NA		NA	NA
	rPO10	mortality	ic	NA	NA			NA		NA	NA
3	rPO1	rate of cure (Measure method: PCR) until PCR negative	ic	NA	NA		j1PO1	The primary endpoint was SARS-CoV-2 negative conversion rate at the 7th day, defined as the percentage of enrolled patients converted to SARS-CoV-2 PCR-negative at the 7th day. The day patients received first dose of honeysuckle decoction or TCM mixture was recorded as 1st day. [supplementary information]	c	NA	NA
4	rPO1	Length of hospital stay	ic	NA	NA		j1PO0	none defined	nd	NA	NA
	rPO2	fever clearance time	ic	NA	NA					NA	NA
5	rPO1	cure rate	ic	p1PO1	The primary end point was the cure rate of the enrolled patients. The definition for cure followed the standard given by	ic	NA	NA		NA	NA

ID		Registry entry (R)	C	p#PO#	Preprint (P)	C	j#PO#	Journal Article (J)	C	Discrepancy rating	
										R vs. P	R vs. J
					the “Diagnosis and Treatment Protocol for Novel Coronavirus Pneumonia (5th or update version)” as 1) fever attenuated for continuously 7 days, 2) twice COVD-19 nucleolus acid detections negative, 3) CT scan shows chest effusion absorbed more than 50% percent when the patient is discharged from hospital						
6	rPO1	declining speed of novel coronavirus by PCR	ic	NA	NA		NA	NA		NA	NA
	rPO2	negative time of novel coronavirus by PCR	ic	NA	NA		j1PO1	In the current study, the time of viral clearance was introduced as a primary endpoint to evaluate the antiviral effect of FPV on the SARS-CoV-2 and successfully identify the priority of FPV; Both FPV and LPV/RTV were continued until the viral clearance was confirmed or until 14 d had passed; ‘’Viral clearance” was defined as the presence of two consecutive negative results with qPCR detection over an interval of 24 h (...) The efficacy of the treatment was assessed by the time of viral clearance and the improvement rate of chest computed tomography (CT) scans on Day 14 after treatment.	c	NA	NA
	rPO3	incidence rate of chest imaging	ic	NA	NA			*[improvement rate of chest computed tomography (CT) scans on Day 14 after treatment.]*		NA	NA
	rPO4	incidence rate of liver enzymes	ic	NA	NA			*[adverse reactions]*		NA	NA
	rPO5	incidence rate of kidney damage	ic	NA	NA			*[adverse reactions]*		NA	NA

ID		Registry entry (R)	C	p#PO #	Preprint (P)	C	j#PO#	Journal Article (J)	C	Discrepancy rating	
										R vs. P	R vs. J
7	rPO1	The time when the nucleic acid of the novel coronavirus turns negative	ic	p1PO0	none defined	nd	NA	NA		NA	NA
	rPO2	T cell recovery time	ic				NA	NA		NA	NA
8	rPO1	Time to viral negativity by RT-PCR	ic	p1PO1	The primary efficacy end point was the percentage of subjects with viral negative by Day 14; SARS-CoV-2 (molecular viral load) was immediately assessed at the hospital laboratory using a semi-quantitative RT-PCR assay	c	j1PO1	The primary efficacy endpoint was the percentage of subjects with viral negative by Day 14; SARS-CoV-2 (molecular viral load) was immediately assessed at the hospital laboratory using a semi-quantitative RT-PCR assay	c	NA	NA
	rPO2	Time to clinical improvement	ic	p1PO2	and the time from randomization to clinical improvement, defined as the time from randomization to an improvement of two points (from the status at randomization) on a seven-category ordinal scale or live discharge from the hospital, whichever came first.	c	j1PO2	and the time from randomization to clinical improvement, defined as the time from randomization to an improvement of two points (from the status at randomization) on a seven-category ordinal scale or live discharge from the hospital, whichever came first.	c	NA	NA
9	rPO1	CT of lung	ic	p1PO1	The primary end point was the CT-based radiological pulmonary changes from baseline to 7 days. *[The secondary end points were [...] radiological pulmonary changes during 7 days to 14 days after treatment.]*	ic	NA	NA		NA	NA
	rPO2	CT and MRI of hip	ic		NA		NA	NA		NA	NA
10	rPO1	critically ill patients (%)	ic	p1PO1	The primary outcome was the change in the disease severity category of COVID-19 after treatment. The severity of COVID-19 was assessed based on the Six-Point Clinical Status Scale for COVID-19 (COVID-19 severity scale); The primary outcome was the change in the disease severity category of COVID-19 after treatment at 7 days	c	j1PO1	The primary outcome was the change in the disease severity category of COVID-19 **after treatment**. The severity of COVID-19 was assessed based on the Six-Point Clinical Status Scale for COVID-19 (COVID-19 severity scale) (Box 1); Nurses **administered** 200 mL of the decoction to patients orally (via feeding tube if needed) twice daily for a total	c	NA	NA

ID		Registry entry (R)	C	p#PO#	Preprint (P)	C	j#PO#	Journal Article (J)	C	Discrepancy rating	
										R vs. P	R vs. J
								of **7 days** in the CHM plus group.			
11	rPO1	The days from positive to negative for viral nucleic acid testing	ic	NA	NA		j1PO1	The primary endpoint was the duration of viral shedding, which was defined as the time from randomization to the first negative nucleic acid test of 5 consecutive RT-PCR results; Discharge criteria were as follows [16]: having a normal temperature for >3 days, significant improvements of respiratory symptoms and CT imaging, and nucleic acid tests negative twice consecutively with an interval of ≥24 hours. After discharge, the patients were isolated at a designated place for 14 days as recommended [16], which was arranged by community committees where the patients resided. They were followed up by primary healthcare facilities and were retested for viral nucleic acid on days 7 and 14. After that, they stayed in their homes for a second isolation period of 14 days and were retested for viral nucleic acid by the end of this quarantine period. We collected each patient’s medical information during the isolation, which was shared with permission. In our study, enrolled patients with 5 consecutively negative nucleic acid tests were considered as having “true negative” results (2 times during hospitalization, 2 times during the first isolation, and 1 time at the end of the second quarantine). If any patient at any time point had a positive test for SARS-CoV-2, they were sent	c	NA	NA

ID		Registry entry (R)	C	p#PO#	Preprint (P)	C	j#PO#	Journal Article (J)	C	Discrepancy rating	
										R vs. P	R vs. J
								to a designated site for isolation and medical observation.			
	NA	NA		NA	NA		j2PO1	Clinical endpoint: the primary endpoint was the time from Leflunomide initiation to clinical improvement meeting the discharge criteria (set by the National Health Commission of China). The discharge criteria include: (1) the body temperature returns to normal for more than 3 days; (2) respiratory symptoms improve significantly; (3) lung imaging shows that acute ostonic lesions significantly improved; (4) two consecutive sputum, nasopharyngeal swabs and other respiratory samples nucleic-acid test are negative (sampling time at least 24 h interval).	ic	NA	NA
12	rPO1	clinical recovery time (28 days)	ic	p1PO1	The primary outcome measurement for this study was time to clinical recovery (TTCR), defined as the number of days from randomization to clinical recovery. Maximum TTCR was 28 days. Patients were considered to have achieved clinical recovery when they had met all of the following criteria for at least 48 hours: 1. axillary body temperature ≤36.9°C or oral body temperature ≤37.2°C; 2. complete relief of all symptoms other than cough; 3. cough graded as mild or absent on a patient-reported scale of severe, moderate, mild, absent.	c	NA	NA		NA	NA
13	rPO1	The changes of clinical symptom, laboratory and	ic	NA	NA			*[The second endpoints were the improvement of clinical*		NA	NA

ID		Registry entry (R)	C	p#PO#	Preprint (P)	C	j#PO#	Journal Article (J)	C	Discrepancy rating	
										R vs. P	R vs. J
		radiological data (Oxyhemoglobin saturation, dyspnea, Body temperature, Radiological characteristic sign, Blood routine, C-reaction protein, lymphocyte count, Liver function: TBIL(total bilirubin), AST(alanine aminotransferase) and ALT(aspartate aminotransferase), Neutralization antibody level): 3 days after convalescent plasma transfusion						*symptoms and laboratory and radiological parameters within 3 d after CP transfusion. Clinical symptoms improvement was defined as temperature normalization, relief of dyspnea, and oxygen saturation normalization, and radiological improvement was defined as different degrees of absorption of lung lesions.]*			
	NA	NA		NA	NA		j1PO1	The primary endpoint was the safety of CP transfusion.	ic	NA	NA
14	rPO1	Clinical symptoms	ic	NA	NA			*[Secondary outcomes included [...], the proportions of patients progressing to critical conditions (eg, acute respiratory distress syndrome, sepsis, or septic shock), [...]]*		NA	NA
	rPO2	Blood routine	ic	NA	NA					NA	NA
	rPO3	the viral load of 2019-nCOV of throat swab	ic	NA	NA			*[Secondary outcomes included [...] viral loads (reflected by the Ct values as measured with real-time RT-PCR assays) at day 21. [...]]*		NA	NA
	rPO4	TBNK cell subsets	ic	NA	NA			*[Secondary outcomes included lymphocyte cell counts on day 5 posttreatment, [...]]*		NA	NA
	rPO5	TH1/TH2 Cytokine	ic	NA	NA					NA	NA
	rPO6	Chest CT	ic	NA	NA					NA	NA
	NA	NA		NA	NA		j1PO1	The primary end point was the time to clinical improvement, ie, the duration from randomization to the improvement of at least 1 point on a 7-category ordinal scale (adopted from a scale for hospitalized patients with severe influenza) or discharge from hospital, whichever	c	NA	NA

ID		Registry entry (R)	C	p#PO#	Preprint (P)	C	j#PO#	Journal Article (J)	C	Discrepancy rating	
										R vs. P	R vs. J
								occurred first. The 7-category ordinal scale was graded based on the following scheme: 1 for nonhospitalized with normal activities; 2 for nonhospitalized but unable to resume normal activities; 3 for hospitalized but not requiring supplemental oxygen; 4 for hospitalized and requiring supplemental oxygen; 5 for hospitalized and requiring nasal high-flow oxygen therapy, noninvasive mechanical ventilation, or both; 6 for hospitalized and requiring extracorporeal membrane oxygenation, invasive ventilation, or both; and 7 for death; Patients were dynamically monitored daily by trained physicians. Diary cards were dispensed to investigators to collect a 7-category ordinal scale and safety score from day 0 to day 21, hospital discharge, or death.			
15	rPO1	Time to Clinical recovery	ic	NA	NA		j1PO1	The primary endpoint was time to clinical improvement, defined as the days from randomization until normalization. Clinical improvement was assessed by five components including body temperature, respiratory rate, oxygen saturation, alleviation of cough, and absorption of pulmonary infection by chest CT. Normalization was defined as body temperature < 37.0 °C, respiratory rate < 24 times per minute indoors, and oxygen saturation > 94% (fingertip). Alleviation of cough was defined as a reduced severity	c	NA	NA

ID		Registry entry (R)	C	p#PO #	Preprint (P)	C	j#PO#	Journal Article (J)	C	Discrepancy rating	
										R vs. P	R vs. J
								of cough from a physician-reported scale of severe or moderate to mild condition or absence. Absorption of pulmonary infection was defined as an absorption area > 2/3 by Digital Imaging and Communications in Medicine (DICOM) images on chest CT. Alleviation of these five clinical symptoms were required to stay normal for at least 72 h on all components to fulfill the primary endpoint of clinical improvement; The primary outcome was set as the time to clinicalimprovement, defined as normalization of body temperature, respiratory rate, oxygen saturation, cough,and absorption of pulmonary infection by chest computed tomography (CT) until 28 d after randomization.			
16	rPO1	Improved respiratory system function (blood oxygen saturation) recovery time	ic	NA	NA		j1PO0	none defined	nd	NA	NA
17	rPO1	SOFA	ic	p1PO1	The primary outcome of this study was the SOFA Score; During the clinical follow-up period (days 1, 2, 3, 4, 5, 6, 7), the following data were collected from patients each day: [...] SOFA score, [...].	c	NA	NA		NA	NA
18	rPO1	2019 nCoV nucleic acid detection [Time Frame: 14 days] Virological clearance rate using Real-Time PCR in upper and/or lower respiratory tract samples at day 3, day 7 and day 14 respectively.	c	p1PO1	The primary study endpoint was the virological clearance (i.e. negative conservation **rate** and **time** to negative) using qRT-PCR in nasopharyngeal swabs samples; Efficacy and safety were assessed at baseline, every day after day 1 to day 14, and every week	c	NA	NA		Change in PO definition	NA

ID		Registry entry (R)	C	p#PO#	Preprint (P)	C	j#PO#	Journal Article (J)	C	Discrepancy rating	
										R vs. P	R vs. J
					thereafter up to day 28 or discharge.						
19	rPO1	Lung CT	ic	p1PO0	none defined	nd	NA	NA		NA	NA
				p2PO0	none defined	nd	NA	NA		NA	NA
20	rPO1	COVID-19 nucleic acid detection time from positive to negative (respiratory secretion) or (3,5,7,10 days from positive to negative rate)	ic	NA	NA		j1PO1	The primary outcome measure was the time required for a negative SARS-CoV-2 RNA result or the negative result rate on day 10; RT-PCR results of blood or respiratory secretion samples	c	NA	NA
	rPO2	Lung CT observation of inflammation absorption	ic	NA	NA		NA	NA		NA	NA
	rPO3	Proportion of cases with progressive disease	ic	NA	NA		NA	NA		NA	NA
21	rPO1	Tempreture	ic	NA	NA		NA	NA		NA	NA
	rPO2	Virus nucleic acid detection	ic	p1PO1	The primary outcome is the time for viral NAA test turning negative (two consecutive negative tests for viral nucleic acid with an interval time not less than 24 hours) after convalescent plasma transfusion	ic	NA	NA		NA	NA
22	rPO1	the number of days between randomised grouping and clinical improvement (Clinical improvement is defined as the patient's admission status of 6 grade scale score reduced by 2 points or discharged.); within 28 days admission	c	NA	NA		j1PO1	The primary end point was time to clinical improvement within a 28-day period. Clinical improvement was defined as patient discharge or a reduction of 2 points on a 6-point disease severity scale. The scale was defined as follows: 6 points, death; 5 points, hospitalization plus extracorporeal membrane oxygenation (ECMO) or invasive mechanical ventilation; 4 points, hospitalization plus noninvasive ventilation or high-flow supplemental oxygen; 3 points, hospitalization plus	c	NA	no discrepancy

ID		Registry entry (R)	C	p#PO#	Preprint (P)	C	j#PO#	Journal Article (J)	C	Discrepancy rating	
										R vs. P	R vs. J
								supplemental oxygen (not high-flow or noninvasive ventilation); 2 points, hospitalization with no supplemental oxygen; 1 point, hospital discharge.			
23	rPO1	Clinical improvement time of 28 days after randomization (The 7-point scale: 7 points: death, 6 points: admission to ECMO and / or mechanical ventilation, 5 points: Hospitalized for non-invasive ventilation and / or high-flow oxygen therapy, 4 points: hospitalization for oxygen therapy, 3 points: Hospitalization does not require oxygen therapy, 2 points: discharged but not restored to normal functional status, 1 point: discharged to normal function)	c	NA	NA		j1PO1	The primary end point was the time to clinical improvement, defined as the time from randomization to an improvement of two points (from the status at randomization) on a seven-category ordinal scale or live discharge from the hospital, whichever came first; The seven-category ordinal scale consisted of the following categories: 1, not hospitalized with resumption of normal activities; 2, not hospitalized, but unable to resume normal activities; 3, hospitalized, not requiring supplemental oxygen; 4, hospitalized, requiring supplemental oxygen; 5, hospitalized, requiring nasal high-flow oxygen therapy, noninvasive mechanical ventilation, or both; 6, hospitalized, requiring ECMO, invasive mechanical ventilation, or both; and 7, death; Patients were assessed once daily by trained nurses using diary cards that captured data on a sevencategory ordinal scale and on safety from day 0 to day 28, hospital discharge, or death.	c	NA	Change in PO definition
24	rPO1	Complete Blood Count	ic	p1PO0	none defined	nd	j1PO0	none defined	nd	NA	NA
	rPO2	CRP	ic							NA	NA
	rPO3	blood coagulation	ic							NA	NA
	rPO4	D-dimer	ic							NA	NA

ID		Registry entry (R)	C	p#PO#	Preprint (P)	C	j#PO#	Journal Article (J)	C	Discrepancy rating	
										R vs. P	R vs. J
	rPO5	virological examination of pharyngeal swab	ic							NA	NA
	rPO6	pulmonary imaging	ic							NA	NA
25	rPO1	nuclear acid test of faeces	ic	p1PO0	none defined	nd	NA	NA		NA	NA
	rPO2	nuclear acid test of the upper respiratory tract	ic							NA	NA
	rPO3	IgM	ic							NA	NA
	rPO4	IgG	ic							NA	NA
	rPO5	CT test of lung	ic							NA	NA
	rPO6	glutamic oxalacetic transaminase	ic							NA	NA
	rPO7	glutamic-pyruvic transaminase	ic							NA	NA
	rPO8	total bilirubin	ic							NA	NA
	rPO9	direct bilirubin	ic							NA	NA
	rPO10	urea nitrogen	ic							NA	NA
	rPO11	lactic dehydrogenase	ic							NA	NA
	rPO12	creatinine	ic							NA	NA
	rPO13	lymphocyte	ic							NA	NA
	rPO14	C-reactive protein	ic							NA	NA
	rPO15	procalcitonin	ic							NA	NA
	rPO16	erythrocyte sedimentation rate	ic							NA	NA
26	rPO1	Clinical recovery rate of day 7	ic	p1PO1	The primary outcome was the clinical recovery rate at 7 days from the beginning of treatment. Clinical recovery was defined as continuous (>72 hours) recovery of body temperature, respiratory rate, oxygen saturation and cough relief after treatment, with following quantitative criteria: axillary temperature ≤36.6°C; respiratory frequency ≤24 times/min; Oxygen saturation ≥98% without oxygen inhalation; mild or no cough.	c	NA	NA		NA	NA
27	rPO1	Viral nucleic acid test	ic	p1PO1	The primary outcome for this trial was whether patients had	c	j1PO1	The primary outcomes for this trial were whether patients had	c	NA	NA

ID		Registry entry (R)	C	p#PO#	Preprint (P)	C	j#PO#	Journal Article (J)	C	Discrepancy rating	
										R vs. P	R vs. J
					a negative conversion of SARS–CoV–2 by 28 days [*and whether patients with severe COVID–19 had a clinical improvement by 28 days. However, since the trial was stopped early and only 2 patients with severe disease were enrolled, results on clinical improvement are not presented.]* Negative conversion of SARS–CoV–2 was defined as two consecutive confirmation of "Negatives" reported at least 24 hours apart without subsequent report of "Positive" SARS–CoV–2 by the end of the study; RT–PCR			negative conversion of SARS-CoV-2 by 28 days *[and whether patients with severe covid-19 had clinical improvement by 28 days. However, as the trial was stopped early and only two patients with severe disease were enrolled, results on clinical improvement are not presented.]* We defined negative conversion of SARS-CoV-2 as two consecutive reports of a negative result for SARS-CoV-2 at least 24 hours apart without a subsequent report of a positive result by the end of the study. We considered the date of the first negative report as the date of negative conversion.			
28	rPO1	novel coronavirus nucleic acid clearance rate; Measure method: RT-PCR; on day 6 after treatment	c	p1PO1	In this regarding, the primary endpoint for this study was decided as the SARS-CoV-2 clearance rates in COVID-19 patients assessed on day 6 of antiviral treatment; SARS-CoV-2 virus nucleic acids were detected by RT-PCR [...]	c	NA	NA		no discrepancy	NA
29	rPO1	Results of SARS-COV2 virus detection; Day 1, Day 4, Day 7 and Day 14	ic	p1PO1	The primary endpoint was virological clearance at day-6 post-inclusion. [...] SARS-CoV-2 RNA was assessed by real-time reverse transcription-PCR	c	j1PO1	The primary endpoint was virological clearance at day-6 post-inclusion; SARS-CoV-2 RNA was assessed by real-time reverse transcription-PCR	c	NA	NA
30	rPO1	PO2/FiO2	ic	p1PO1	Primary outcomes include PaO2/FiO2; The changes of PaO2/FiO2 and ROX index indicating respiratory function, (...) for the 7th day were retrospectively analyzed.	c	NA	NA		NA	NA
	rPO2	ROX INDEX	ic	p1PO2	ROX index (ROX=SpO2/(FiO2*RR)); The	c	NA	NA		NA	NA

ID		Registry entry (R)	C	p#PO#	Preprint (P)	C	j#PO#	Journal Article (J)	C	Discrepancy rating	
										R vs. P	R vs. J
					changes of PaO2/FiO2 and ROX index indicating respiratory function, (...) for the 7th day were retrospectively analyzed.						
31	rPO1	Prostaglandin E2	ic	p1PO0	none defined	nd	j1PO0	none defined	nd	NA	NA
32	rPO1	Partial arterial oxygen pressure (PaO2) to fraction of inspiration O2 (FiO2) ratio [Time Frame: 24 hours] Partial arterial oxygen pressure (PaO2) to fraction of inspiration O2 (FiO2) ratio	c	p1PO1	Primary outcomes were changes of PaO2/FiO2 at day 1 [...]. PaO2 was measured 6 by arterial blood gas (ABG) and FiO2 at the time of clinical ABG was obtained.	c	NA	NA		no discrepancy	NA
	rPO2	Partial arterial oxygen pressure (PaO2) to fraction of inspiration O2 (FiO2) ratio [Time Frame: 7 days] Partial arterial oxygen pressure (PaO2) to fraction of inspiration O2 (FiO2) ratio	c	p1PO2	Primary outcomes were changes of PaO2/FiO2 at [...] day 7. PaO2 was measured 6 by arterial blood gas (ABG) and FiO2 at the time of clinical ABG was obtained.	c				no discrepancy	NA
33	rPO1	new-onset COVID-19 [Time Frame: From date of randomization until the diagnosis of COVID-19, assessed up to 6 weeks.] new-onset coronavirus disease-2019	ic	p1PO1	The primary outcome was the development of COVID-19 pneumonia by the 28th day after the preventive drug intervention. (...) Diagnosis was made according to the “Diagnosis and Treatment Protocol for Novel Coronavirus Pneumonia” issued by the General Office of the National Health Commission of the People’s Republic of China [9], as follows:<br><br>SARS-CoV-2 infection: Positive pathogenic test (nucleic acid or specific antibody), including:	c	NA	NA		NA	NA
34	rPO1	Rate of composite adverse outcomes [Time Frame: 14 days] Defined as SPO2≤ 93% without oxygen supplementation, PaO2/FiO2 ≤300mmHg or a respiratory	c	p1PO0	none defined	nd	j1PO1	Primary endpoint is the rate of composite adverse outcomes (defined as SPO2 ≤ 93% without oxygen supplementation, PaO2/FiO2 ≤300 mm Hg or a respiratory	ic	no PO defined	Change in PO definition

ID		Registry entry (R)	C	p#PO#	Preprint (P)	C	j#PO#	Journal Article (J)	C	Discrepancy rating	
										R vs. P	R vs. J
		rate ≥30 breaths per min without supplemental oxygen						rate ≥30 breaths per min without supplemental oxygen).			
35	rPO1	Clinical cure rate [Time Frame: 3 months] Definition of clinical cure: The viral load of the respiratory specimen was negative for two consecutive times (the interval between the two tests was greater than or equal to one day), the lung image improved, and the body temperature returned to normal for more than 3 days, and the clinical manifestation improved.	ic	NA	NA			NA		NA	NA
	NA	NA		NA	NA		j1PO1	The primary outcome was the cumulative lung lesion remission rate (lung CT examination indicated absorption of lung inflammation).	ic	NA	NA
36	rPO1	arrest in deterioration of pulmonary function [Time Frame: 7days] rate of patients with no need in increase of FiO2 to maintain stable SO2 and no need of intubation	c	p1PO0	none defined	nd	NA	NA		no PO defined	NA
	rPO2	improving in pulmonary function [Time Frame: 7 days] rate of patients with change of oxygen saturation >3 percentage points or >10% or decrease in FiO2 need or reduction in pulmonary consolidations >30% at HR CT-scan	c	p2PO1	The primary outcome was the rate of responder patients. A patient was a-priori defined as responder if fulfilling either criteria 1 or 2 AND criteria 3 of the ones listed below:<br>1) Improvement of oxygen saturation by more than 3% points and/or increase in P/F by 50% and/or increase P/F above 150 mmHg 72 hours after tocilizumab AND persistence of this improvement at day 7;<br>2) No worsening of respiratory function as defined in the inclusion criteria at 72 hours AND improvement of oxygen saturation by more than 3%	c	NA	NA		Change in PO definition	NA

ID		Registry entry (R)	C	p#PO#	Preprint (P)	C	j#PO#	Journal Article (J)	C	Discrepancy rating	
										R vs. P	R vs. J
					points and/or increase in P/F>50% and/or increase P/F above 150 mmHg at day 7;<br>3) No need of endotracheal ventilation for all or CP for those not requiring it at baseline<br><br>Complete clinical evaluation, assessment of SO2, arterial blood gas analysis (when possible), recording of both FiO2 and type of ventilation were performed at baseline, and after tocilizumab infusion at 24 and 72 hours and day 7. Complete blood count, ALT, D-dimer, and creatinine. were also performed at the same time points.						
37	rPO1	Lethality rate two weeks after registration [Time Frame: up to 15 days] 2-week lethality is defined as the ratio of the number of subjects dead within 14 days from study start out of phase 2 patients with baseline information.	c	p1PO1	The primary aim of the study was to estimate lethality rates at 14 [...] days in the ITT phase 2 population.	c	j1PO1	14-day lethality rate	c	no discrepancy	no discrepancy
	rPO2	Lethality rate one month after registration [Time Frame: up to 1 month] 1-month lethality is defined as the ratio of the number of subjects dead within 30 days from study start out of phase 2 patients with baseline information.	c	p1PO2	The primary aim of the study was to estimate lethality rates at [...] 30-days in the ITT phase 2 population.	c	j1PO2	30-day lethality rate	c	no discrepancy	no discrepancy
38	rPO1	Clinical symptoms (fever, weakness, cough) recovery rate	ic	NA	NA		j1PO1	The primary endpoint was the rate of symptom (fever, fatigue, and coughing) recovery. Fever denoted the subaxillary temperature being 37.3 degrees or greater. The magnitude of fatigue and coughing was self-reported by the patients. Recovery of	c	NA	NA

ID		Registry entry (R)	C	p#PO#	Preprint (P)	C	j#PO#	Journal Article (J)	C	Discrepancy rating	
										R vs. P	R vs. J
								symptoms was defined as the complete resolution of fever, fatigue and coughing; Vital signs, laboratory testing, chest computed tomography and nucleic acid assays of SARS-CoV-2 were evaluated at baseline after randomization and on day 14.			
	rPO2	Clinical symptoms (fever, weakness, cough) recovery time	ic					*[Secondary endpoints consisted of the time to symptom recovery, the rate of and the time to the recovery of individual symptoms, the proportion of patients with improvement on chest computed tomography, the proportion of patients with clinical cure, the timing and rate of conversion of SARS-CoV-2 RNA assay.; Fever denoted the subaxillary temperature being 37.3 degrees or greater. The magnitude of fatigue and coughing was self-reported by the patients. Recovery of symptoms was defined as the complete resolution of fever, fatigue and coughing.]*		NA	NA
39	rPO1	Response to the treatment (According the clinical, paraclinical and laboratory findings); Daily	ic	p1PO1	Primary outcome of study was time to reach clinical response. Clinical response was defined according to the six-category ordinal scale [19]. This scale classifies patients in six categories according to the severity of the viral pneumonia. The six categories are: (1) discharge (2) hospital admission, not requiring oxygen (3) hospital admission, requiring oxygen (4) hospital admission, requiring non-invasive positive pressure ventilation (5) hospital admission requiring invasive	c	j1PO1	The primary outcome of the study was time to reach clinical response. Clinical response was defined according to the six-category ordinal scale (47). This scale classifies patients into six categories according to the severity of the viral pneumonia: (1) discharge; (2) hospital admission, not requiring oxygen; (3) hospital admission, requiring oxygen; (4) hospital admission, requiring noninvasive positive pressure ventilation; (5) hospital admission, requiring invasive mechanical	c	NA	NA

ID		Registry entry (R)	C	p#PO#	Preprint (P)	C	j#PO#	Journal Article (J)	C	Discrepancy rating	
										R vs. P	R vs. J
					mechanical ventilation (6) death. Time to clinical response was considered days required to at least two scores improvement in the scale or patient's discharge, which one that occurred sooner; The duration of study was two weeks and the patients were followed up to four weeks.			ventilation; (6) death. Time to clinical response was considered the number of days required to at least two scores of improvement on the scale or patient's discharge, whichever occurred sooner; The duration of the study was 2 weeks, and the patients were monitored for 4 weeks; Patients were assessed to fit in one of the six categories of the ordinal scale at days 0, 7, 14, and 28 of inclusion. If discharged, the patient was followed up by phone. Readmission was surveyed until 3 May.			
	rPO2	Complications of the treatment (Interview and patient's record); Daily	ic		*[Secondary outcomes duration of hospital stay, length of ICU stay, 28-day mortality, effect of early or late administration of IFN on mortality, adverse effects and complications during the hospitalization. Following adverse effects of the antiviral regimen/IFN β-1a and complications during the hospitalization course were assessed: gastrointestinal (nausea, vomiting, diarrhea, abdominal pain, pancreatitis), anaphylaxis and allergic reactions (rash, urticaria, angioedema, bronchospasm, and dyspnea related to medication administration), IFN injection-related reaction (skin erythema and necrosis, chills, fever, and flu-like symptoms after injection), neuropsychiatric (sleep disorder, psychosis, agitation, depression, and mania), renal impairment (according to KDIGO definition) [17], hepatic impairment (hepatic aminotransferases*			*[Secondary outcomes were duration of mechanical ventilation, duration of hospital stay, length of ICU stay, 28-day mortality, effect of early or late (before or after 10 days of the onset of symptoms) administration of IFN on mortality, adverse effects, and complications during the hospitalization. The following adverse effects of the antiviral regimen/IFN -1a and complications during the hospitalization course were assessed: gastrointestinal (nausea, vomiting, diarrhea, abdominal pain, and pancreatitis), anaphylaxis and allergic reactions (rash, urticaria, angioedema, bronchospasm, and dyspnea related to medication administration), IFN injection-related reaction (skin erythema and necrosis, chills, fever, and flu-like symptoms after injection), neuropsychiatric (sleep disorder, psychosis,*		NA	NA

ID		Registry entry (R)	C	p#PO#	Preprint (P)	C	j#PO#	Journal Article (J)	C	Discrepancy rating	
										R vs. P	R vs. J
					serum levels raised more than three times the upper limit of normal or serum total bilirubin above 2 mg/dL) [20], Indirect hyperbilirubinemia (direct bilirubin level less than 15% of the total bilirubin) [21], incidence of thromboembolism (deep vein thrombosis or pulmonary thromboembolism), incidence of nosocomial infections, diagnosis of septic shock (according to surviving sepsis campaign guideline) [22]. The Naranjo scale was used for evaluation of adverse effects of IFN [23].]			*agitation, depression, and mania), renal impairment (according to KDIGO definition) (46), hepatic impairment (hepatic aminotransferase serum levels raised more than three times the upper limit of normal or serum total bilirubin above 2 mg/dl) (48), indirect hyperbilirubinemia (direct bilirubin level less than 15% of the total bilirubin) (49), incidence of thromboembolism (deep-vein thrombosis or pulmonary thromboembolism), incidence of nosocomial infections, and diagnosis of septic shock (according to the surviving sepsis campaign guidelines) (50). The Naranjo scale was used for evaluation of adverse effects of IFN. In this standard scale, several items, including previous reports, relationship with starting the agent, improvement after discontinuation, challenge result, alternative causes, data of drug assay, dose dependency, and patient's history of the same reaction were considered. Total scores of 8, 5 to 8, and 1 to 4 were considered definite, probable, and possible correlations between the use of IFN and the adverse drug reaction, respectively (51).]*			
40	rPO1	clinical response to therapy; daily	ic	NA	NA		j1PO1	Primary outcome was the symptoms remission. Fever, cough, dyspnea, myalgia, malaise, rhinorrhea, arthralgia, chest pain, headache, vomiting, diarrhea, and sore throat were evaluated every day for 14 days. Laboratory	ic	NA	NA

ID		Registry entry (R)	C	p#PO#	Preprint (P)	C	j#PO#	Journal Article (J)	C	Discrepancy rating	
										R vs. P	R vs. J
								results measured at baseline and at days 7 and 14.			
41	rPO1	CT-scan findings (Method of measurement: CT-scan); Timepoint: Before the intervention and 2 weeks after the intervention	ic	NA	NA			*[Secondary outcomes were clinical improvements (e.g., resolution of fever, cough and dyspnea) and improvement of CT findings at days 14 after initiation of the treatment.]*		NA	NA
	NA	NA		NA	NA		j1PO1	The primary outcome of this study was the rate of hospitalization	ic	NA	NA
42	rPO1	Time to Clinical Improvement (TTCI) [Censored at Day 28] [Time Frame: up to 28 days] The primary endpoint is time to clinical improvement (censored at Day 28), defined as the time (in days) from randomization of study treatment (remdesivir or placebo) until a decline of two categories on a six-category ordinal scale of clinical status (1 = discharged; 6 = death) or live discharge from hospital. Six-category ordinal scale: 6. Death; 5. ICU, requiring ECMO and/or IMV; 4. ICU/hospitalization, requiring NIV/ HFNC therapy; 3. Hospitalization, requiring supplemental oxygen (but not NIV/ HFNC); 2. Hospitalization, not requiring supplemental oxygen; 1. Hospital discharge or meet discharge criteria (discharge criteria are defined as clinical recovery, i.e. fever, respiratory rate, oxygen saturation return to normal, and cough relief). Abbreviation: IMV, invasive mechanical ventilation; NIV, non-invasive mechanical ventilation; HFNC, High-flow nasal cannula.	c	NA	NA		j1PO1	The primary clinical endpoint was time to clinical improvement within 28 days after randomisation. Clinical improvement was defined as a two-point reduction in patients' admission status on a six-point ordinal scale, or live discharge from the hospital, whichever came first. The six-point scale was as follows: death=6; hospital admission for extracorporeal membrane oxygenation or mechanical ventilation=5; hospital admission for non-invasive ventilation or high-flow oxygen therapy=4; hospital admission for oxygen therapy (but not requiring high-flow or non-invasive ventilation)=3; hospital admission but not requiring oxygen therapy=2; and discharged or having reached discharge criteria (defined as clinical recovery—ie, normalisation of pyrexia, respiratory rate <24 breaths per minute, saturation of peripheral oxygen >94% on room air, and relief of cough, all maintained for at least 72 h)=1. The six-point scale was	c	NA	no discrepancy

ID		Registry entry (R)	C	p#PO#	Preprint (P)	C	j#PO#	Journal Article (J)	C	Discrepancy rating	
										R vs. P	R vs. J
								modified from the seven-point scale used in our previous COVID-19 lopinavir–ritonavir RCT by combining the two outpatient strata into one.			
43	rPO1	Time to negative NPS [Time Frame: Up to 1 month] Time to negative NPS 2019-n-CoV RT-PCR	c	NA	NA		j1PO1	The primary endpoint was time to achieve a negative RT-PCR result for SARS-CoV-2 in a nasopharyngeal swab sample; All patients were followed up at the infectious disease clinic within 30 days after discharge	c	NA	no discrepancy
44	rPO1	To assess the safety of baricitinib combined with antiviral (lopinavir-ritonavir) in terms of serious or non-serious adverse events incidence rate. [Time Frame: 2 weeks] All adverse event recording	c	NA	NA		j1PO0	none defined	nd	NA	no PO defined
45	rPO1	Rate of aggravation [Time Frame: within 14 days from the start of medication] Aggravation was defined as(one of them): respiratory distress, RR ≥ 30 times / min; SpO2 ≤ 93% in resting state; arterial partial pressure of oxygen (PaO2) /concentration of oxygen (FiO2) ≤ 300mmHg	c	NA	NA		j1PO1	The primary end points were [...] the deterioration rate after initiation of medications. [...] Disease deterioration was defined as the presence of respiratory distress, respiratory rate ≥ 30 times/minute, oxygen saturation ≤ 93% in the resting state, and oxygenation index ≤ 300 mmHg; during the study period of 14 days.	c	NA	no discrepancy
	rPO2	Time to clinical recovery after treatment [Time Frame: within 14 days from the start of medication] Defined as random to fever, respiratory rate return to normal and cough remission over 48 hours.	ic	NA	NA		j1PO2	The primary end points were the time to clinical recovery [...] Clinical recovery was defined as clinical symptoms (fever and respiratory symptoms) returning to normal over 48 hours; during the study period of 14 days.	ic	NA	NA
46	rPO1	P/F ratio [Time Frame: At baseline and 24, 48 and 168 hours after treatment initiation] Change in ratio between partial pressure of oxygen in arterial blood, measured by means of	c	NA	NA		j1PO1	change in PaO2/FiO2 ratio; after 24, 48 and 168 hours	c	NA	no discrepancy

ID		Registry entry (R)	C	p#PO#	Preprint (P)	C	j#PO#	Journal Article (J)	C	Discrepancy rating	
										R vs. P	R vs. J
		arterial blood gas analysis, and inspired oxygen fraction at baseline and after study treatment									
	rPO2	PaO2 difference [Time Frame: At baseline and 24, 48 and 168 hours after treatment initiation] Change in partial pressure of oxygen in arterial blood, measured by means of arterial blood gas analysis, at baseline and after study treatment	c	NA	NA		j1PO2	change in PaO2; after 24, 48 and 168 hours	c	NA	no discrepancy
	rPO3	A-a O2 difference [Time Frame: At baseline and 24, 48 and 168 hours after treatment initiation] Change in alveolar-arterial gradient of oxygen at baseline and after study treatment. Arterial alveolar gradient will be calculated using the following parameters derived from arterial blood gas analysis: partial pressure of oxygen in arterial blood and partial pressure of carbon dioxide in arterial blood.	c	NA	NA		j1PO3	change in alveolar-arterial oxygen (A-a O2); after 24, 48 and 168 hours	c	NA	no discrepancy
47	rPO1	Combination of death, ICU stay or non-invasive ventilation (NIV); At the time of discharge or death	ic	p1PO1	The primary outcome measure was a composite endpoint that included in-hospital all-cause mortality, escalation to ICU admission, or progression of respiratory insufficiency that required non-invasive ventilation (NIV); Patients were censored at hospital discharge or day 15 after inclusion.	ic	NA	NA		NA	NA
48	rPO1	Improved clinical status [Time Frame: Day 14] Percent of subjects with improved clinical status	ic	NA	NA		j1PO1	Co-primary endpoints were [...] improved clinical severity defined by mild, moderate, severe, or critical COVID-19 (6) by day 14.<br>[for definition of clinical severity, please see references: 6. China National	c	NA	NA

ID		Registry entry (R)	C	p#PO#	Preprint (P)	C	j#PO#	Journal Article (J)	C	Discrepancy rating	
										R vs. P	R vs. J
								Health Commission: Chinese Clinical Guidance for COVID19 Pneumonia Diagnosis and Treatment (7th Edition). Available at: http://kjfy. meetingchina.org/msite/news/show/cn/3337.html. Accessed March 4, 2020]			
	rPO2	Return to room air [Time Frame: Day 14] Percent of subjects return to room air	c	NA	NA		j1PO2	Co-primary endpoints were return to room air (RTRA) [...] by day 14; resolution of supplemental oxygen need (return to room air [RTRA]) (see Introduction)	c	NA	no discrepancy
49	rPO1	Time to Recovery [Time Frame: Day 1 through Day 29] Day of recovery is defined as the first day on which the subject satisfies one of the following three categories from the ordinal scale: 1) Hospitalized, not requiring supplemental oxygen - no longer requires ongoing medical care; 2) Not hospitalized, limitation on activities and/or requiring home oxygen; 3) Not hospitalized, no limitations on activities.	c	NA	NA		j1PO1	The primary outcome was the time to recovery, defined as the first day, during the 28 days after enrollment, on which a patient met the criteria for category 1, 2, or 3 on the eight-category ordinal scale. The categories are as follows: 1, not hospitalized and no limitations of activities; 2, not hospitalized, with limitation of activities, home oxygen requirement, or both; 3, hospitalized, not requiring supplemental oxygen and no longer requiring ongoing medical care (used if hospitalization was extended for infection-control or other nonmedical reasons); 4, hospitalized, not requiring supplemental oxygen but requiring ongoing medical care (related to Covid-19 or to other medical conditions); 5, hospitalized, requiring any supplemental oxygen; 6, hospitalized, requiring noninvasive ventilation or use of high-flow oxygen devices; 7, hospitalized, receiving invasive mechanical ventilation or	c	NA	no discrepancy

ID		Registry entry (R)	C	p#PO#	Preprint (P)	C	j#PO#	Journal Article (J)	C	Discrepancy rating	
										R vs. P	R vs. J
								extracorporeal membrane oxygenation (ECMO); and 8, death.			
50	rPO1	Clinical deterioration in the semiquantitative ordinal scale suggested by the WHO R&D committee [Time Frame: 3 weeks] Time to clinical deterioration (2 levels in the WHO R&D Blueprint scale)	c	NA	NA		j1PO1	The primary end point of the clinical phase was the time from baseline to clinical deterioration, defined as a 2-grade increase on an ordinal clinical scale, based on the World Health Organization R&D Blueprint Ordinal Clinical Scale,9 as used in previously published studies, within a time frame of 3 weeks after randomization or until hospital discharge (whichever occurred first).	c	NA	no discrepancy
	rPO2	Maximal concentration of cardiac troponin [Time Frame: 10 days] Maximal concentration of high-sensitivity cardiac troponin	c	NA	NA		j1PO2	maximal high-sensitivity cardiac troponin (hs cTn) levels between the 2 groups; The maintenance dosage was 0.5 mg colchicine twice daily (reduced to once daily among patients with body weight<60 kg) until hospital discharge or a maximum of 21 days. All patients were observed during this timeframe with daily reassessment of their clinical status and complete laboratory hematologic and biochemical evaluation every 48 hours.	c	NA	Change in PO definition
	NA	NA		NA	NA		j1PO3	time for C-reactive protein to reach levels greater than 3 times the upper reference limit; The maintenance dosage was 0.5 mg colchicine twice daily (reduced to once daily among patients with body weight<60 kg) until hospital discharge or a maximum of 21 days. All patients were observed during this timeframe with daily reassessment of their clinical status and complete laboratory	c	NA	PO added or upgraded from non-primary

ID		Registry entry (R)	C	p#PO#	Preprint (P)	C	j#PO#	Journal Article (J)	C	Discrepancy rating	
										R vs. P	R vs. J
								hematologic and biochemical evaluation every 48 hours.			
51	rPO1	Need of invasive and non invasive mechanical ventilation; daily in all duration of study	ic	NA	NA			NA		NA	NA
	rPO2	need of oxygen supplement; daily in all duration of study	ic	NA	NA			NA		NA	NA
	rPO3	the median time for recovery; daily in all duration of study	ic	NA	NA			NA		NA	NA
	rPO4	well-being; daily in all duration of study	ic	NA	NA			NA		NA	NA
	NA	NA		NA	NA		j1PO1	The primary outcome of the study was length of hospital stay	ic	NA	PO added or upgraded from non-primary
52	rPO1	Time to negatively RT-PCR [Time Frame: 14 days] To evaluate the efficacy of HCQ, with respect to the time to negatively RT-PCR assessments in COVID-19 patients.	c	p1PO1	The primary endpoint was to evaluate the efficacy of HCQ with respect to time to negative rRT-PCR assessments from randomization up to 14 days	c	j1PO1	The primary endpoint was to evaluate the time to negative rRT-PCR assessments from randomization, up to 14 days, by arm.	c	no discrepancy	no discrepancy
53	rPO1	Polymerase chain reaction (PCR) confirmed SARS-CoV-2 infection [Time Frame: Day 1 through Day 14 after enrolment] Polymerase chain reaction (PCR) confirmed SARS-CoV-2 infection from self-collected samples collected daily for 14 days	c	NA	NA		j1PO1	The primary trial end point was reverse-transcription PCR (RT-PCR)–confirmed SARS-CoV-2 infection assessed via samples collected daily through day 14, among participants who were SARS-CoV-2 negative at baseline. To detect viable virus, a sensitivity end point was defined by using a lower PCR cycle threshold (Ct) cutoff of 38 (indicating higher viral load) if a target was positive to define a SARS-CoV-2–positive test.	c	NA	no discrepancy
	rPO2	Polymerase chain reaction (PCR) confirmed SARS-CoV-2 infection [Time Frame: Day 28 after enrolment] Polymerase chain reaction (PCR) confirmed	c	NA	NA			*[The secondary analysis for day 28 outcomes included the day 28 SARS-CoV-2 PCR result and reported positive SARS-CoV-2 tests between day 1 and 28.]*		NA	PO downgraded to secondary

ID		Registry entry (R)	C	p#PO#	Preprint (P)	C	j#PO#	Journal Article (J)	C	Discrepancy rating	
										R vs. P	R vs. J
		SARS-CoV-2 infection from self-collected samples collected at study exit									
54	rPO1	Feasibility of performing study pathway consisting of consenting convalescent donors, harvesting convalescent plasma, application for FDA eIND and administering convalescent plasma to the patients [Time Frame: 28 days after plasma administration] Feasibility will be measured by (number of donors from whom convalescent plasma is harvested/number of interested donors) and number of patients who receive convalescent plasma at day 28.	c	p1PO1	The primary outcome was feasibility as defined by the collection of convalescent plasma and its administration into hospitalized patients.	ic	NA	NA	ic	Change in PO definition	
				p2PO1	The primary outcome was feasibility as defined by the collection of convalescent plasma and its administration into hospitalized patients.	ic	j1PO1	The primary outcome was feasibility as defined by the collection of convalescent plasma and its administration into hospitalized patients.		Change in PO definition	Change in PO definition
	rPO2	Type of respiratory support [Time Frame: 28 days after plasma administration] Levels of respiratory support will be graded (e.g. room air, high flow oxygen, intubation) to determine the change in type of respiratory support at 28 days.	c	NA	*[Secondary outcomes included type and duration of respiratory support, cardiac arrest, transfer to intensive care unit (ICU), length of stay, mortality, complications of plasma administration and process outcomes]*			*[Secondary outcomes included type and duration of respiratory support, cardiac arrest, transfer to intensivecare unit (ICU), length of stay, mortality, complications of plasma administration, process outcomes and antibody titre of plasma donors andrecipients.]*		PO downgraded to secondary	PO downgraded to secondary
				NA	*[Secondary outcomes included type and duration of respiratory support, cardiac arrest, transfer to intensive care unit (ICU), length of stay, mortality, complications of plasma administration and process outcomes]*			NA		PO downgraded to secondary	
55	rPO1	The proportion of patients with improved disease severity at	c	NA	NA		j1PO1	The primary endpoint was the proportion of patients with	c	NA	no discrepancy

ID		Registry entry (R)	C	p#PO#	Preprint (P)	C	j#PO#	Journal Article (J)	C	Discrepancy rating	
										R vs. P	R vs. J
		day 2 [Time Frame: from baseline to day 2] The proportion of patients with improved disease severity (by at least one scale) at day 2						improved disease severity (by at least one scale); Clinical assessments included the five-category ordinal scale [see Panel 1 in Online Supplement (http://dx.doi.org/10.21037/jtd-2020- 057)], four-category ordinary scale of dyspnea, coughing, chest distress and chest pain (0: None; 1: Mild; 2: Moderate; 3: Severe; 4: Very severe) and adverse events, performed on admission, at enrollment, at days 2 [...].			
	rPO2	The proportion of patients with improved disease severity at day 3 [Time Frame: from baseline to day 3] The proportion of patients with improved disease severity (by at least one scale) at day 3	c	NA	NA		j1PO2	The primary endpoint was the proportion of patients with improved disease severity (by at least one scale); Clinical assessments included the five-category ordinal scale [see Panel 1 in Online Supplement (http://dx.doi.org/10.21037/jtd-2020- 057)], four-category ordinary scale of dyspnea, coughing, chest distress and chest pain (0: None; 1: Mild; 2: Moderate; 3: Severe; 4: Very severe) and adverse events, performed on admission, at enrollment, at days [...] 3, [...].	c	NA	no discrepancy
	rPO3	The proportion of patients with improved disease severity at the day before hospital discharge [Time Frame: up to 14 days (from baseline to the day before hospital discharge)] The proportion of patients with improved disease severity (by at least one scale) at the day before hospital discharge	c	NA	NA		j1PO3	The primary endpoint was the proportion of patients with improved disease severity (by at least one scale); Clinical assessments included the five-category ordinal scale [see Panel 1 in Online Supplement (http://dx.doi.org/10.21037/jtd-2020- 057)], four-category ordinary scale of dyspnea, coughing, chest distress and chest pain (0: None; 1: Mild; 2: Moderate; 3: Severe; 4: Very severe) and adverse events, performed on admission, at enrollment, [...] and the day	c	NA	no discrepancy

ID		Registry entry (R)	C	p#PO#	Preprint (P)	C	j#PO#	Journal Article (J)	C	Discrepancy rating	
										R vs. P	R vs. J
								before discharge (end-of-treatment).			
56	rPO1	Safety indexes of adverse reactions [Time Frame: 0-7 days post-vaccination] Occurrence of adverse reactions post-vaccination	c	NA	NA		j1PO1	The primary endpoint for safety was the occurrence of adverse reactions within 7 days after the vaccination; Adverse events were self-reported by the participants, but verified by investigators daily during the first 14 days after vaccination. Subsequently, adverse events were recorded by the participants on diary cards in the following weeks. Laboratory safety tests including white blood cell count, lymphocyte count, neutrophils, platelets, haemoglobin, alanine aminotransferase, aspartate aminotransferase, total bilirubin, fasting blood glucose, and creatinine were measured on day 7 to assess any toxic effects post-vaccination. We graded adverse events and abnormal changes in laboratory tests according to the scale issued by the China State Food and Drug Administration (version 2019).	c	NA	no discrepancy
57	rPO1	Viral clearance [Time Frame: 14 days] Test for virus at day 1 and 14 from beginning of trial drug started	ic	NA	NA		j1PO1	The primary end point was the efficacy of the combination therapy. A negative PCR was counted as treatment success.	ic	NA	NA
58	rPO1	SARS-CoV-2 clearance	ic	p1PO1	The primary outcomes included the rate of SARS-CoV-2 clearance; Two consecutive negative RT-PCR results 24 hours apart for SARS-CoV-2 indicated SARS-CoV-2 clearance; [...] one day before and daily after the start of leflunomide therapy until discharge from the hospital	c	NA	NA		NA	NA

ID		Registry entry (R)	C	p#PO #	Preprint (P)	C	j#PO#	Journal Article (J)	C	Discrepancy rating	
										R vs. P	R vs. J
	NA	NA		p1PO2	time to SARS-CoV-2 clearance; Two consecutive negative RT-PCR results 24 hours apart for SARS-CoV-2 indicated SARS-CoV-2 clearance; [...] one day before and daily after the start of leflunomide therapy until discharge from the hospital	c	NA	NA		NA	NA
	NA	NA		p1PO3	the 14-day [...] hospital discharge rate.	c	NA	NA		PO added or upgraded from non-primary	NA
	NA	NA		p1PO4	and 30-day hospital discharge rate.	c	NA	NA		PO added or upgraded from non-primary	NA
59	rPO1	death [Time Frame: within 7 days] death from any cause	c	p1PO1	Primary endpoint of the study is 7-days mortality	c	j1PO1	The primary endpoint of the study was 7-day mortality	c	no discrepancy	no discrepancy
60	rPO1	Part A: Percentage of Participants in Each Clinical Status Category as Assessed by a 7-Point Ordinal Scale on Day 11 [Time Frame: Day 11] Clinical status was derived from death, hospital discharge, and ordinal scale as follows: score of "1" was used for all days on or after the date of death; score of "7" was used for all days on or after discharged alive date; last available assessment for missing value. The scale is as follows: 1. Death; 2. Hospitalized, on invasive mechanical ventilation or Extracorporeal Membrane Oxygenation (ECMO); 3. Hospitalized, on non-invasive ventilation or high flow oxygen devices; 4. Hospitalized, requiring low flow supplemental oxygen; 5. Hospitalized, not requiring supplemental oxygen	c	NA	NA		j1PO1	The primary efficacy end point was the distribution of clinical status assessed on the 7-point ordinal scale on study day 11	c	NA	no discrepancy

ID		Registry entry (R)	C	p#PO#	Preprint (P)	C	j#PO#	Journal Article (J)	C	Discrepancy rating	
										R vs. P	R vs. J
		- requiring ongoing medical care (coronavirus (COVID-19) related or otherwise); 6. Hospitalized, not requiring supplemental oxygen - no longer required ongoing medical care (other than per protocol remdesivir administration; 7. Not hospitalized. The odds ratio represents the odds of improvement in the ordinal scale for a RDV group relative to the SOC group.									
61	rPO1	Part A: Percentage of Participants in Each Clinical Status Category as Assessed by a 7-Point Ordinal Scale on Day 14 [Time Frame: Day 14] Clinical status was derived from death, hospital discharge, and the ordinal scale as follows: score of "1" was used for all days on or after the date of death; score of "7" was used for all days on or after discharged alive date; last assessment carried forward for any missing values.The scale is as follows: 1. Death; 2. Hospitalized, on invasive mechanical ventilation or ECMO; 3. Hospitalized, on non-invasive ventilation or high flow oxygen devices; 4. Hospitalized, requiring low flow supplemental oxygen; 5. Hospitalized, not requiring supplemental oxygen - requiring ongoing medical care (COVID-19 related or otherwise); 6. Hospitalized, not requiring supplemental oxygen - no longer required ongoing medical care (other than per protocol remdesivir	c	NA	NA		j1PO1	The primary efficacy end point was clinical status assessed on day 14 on a 7-point ordinal scale consisting of the following categories: 1, death; 2, hospitalized, receiving invasive mechanical ventilation or ECMO; 3, hospitalized, receiving noninvasive ventilation or high-flow oxygen devices; 4, hospitalized, requiring low-flow supplemental oxygen; 5, hospitalized, not requiring supplemental oxygen but receiving ongoing medical care (related or not related to Covid-19); 6, hospitalized, requiring neither supplemental oxygen nor ongoing medical care (other than that specified in the protocol for remdesivir administration); and 7, not hospitalized	c	NA	no discrepancy

ID		Registry entry (R)	C	p#PO#	Preprint (P)	C	j#PO#	Journal Article (J)	C	Discrepancy rating	
										R vs. P	R vs. J
		administration); 7. Not hospitalized. The odds ratio represents the odds of improvement in the ordinal scale for the Remdesivir for 10 days group vs the Remdesivir for 5 days group.									
62	rPO1	Evaluation of the clinical status [Time Frame: 15 days after randomization] Evaluation of the clinical status of patients on the 15th day after randomization defined by the Ordinal Scale of 7 points.<br><br>Alive at home without limitations on activities<br>Alive at home without limitations on activities<br>In the hospital without oxygen<br>In the hospital using oxygen<br>In the hospital using high-flow nasal catheter or non-invasive ventilation<br>In hospital, on mechanical ventilation<br>Dead	c	NA	NA		j1PO1	The primary outcome was clinical status at 15 days, evaluated with the use of a seven-level or-dinal scale. Scores on the scale were defined as follows: a score of 1 indicated not hospitalized with no limitations on activities; 2, not hospital-ized but with limitations on activities; 3, hospital-ized and not receiving supplemental oxygen; 4, hospitalized and receiving supplemental oxygen; 5, hospitalized and receiving oxygen supplemen-tation administered by a high-f low nasal cannula or noninvasive ventilation; 6, hospitalized and receiving mechanical ventilation; and 7, death.	c	NA	no discrepancy
63	rPO1	"COVID Ordinal Outcomes Scale at 14 days [Time Frame: Assessed once on day 14 after enrollment (enrollment is day 0)] Per https://www.who.int/blueprint/priority-diseases/key-action/COVID-19_Treatment_Trial_Design_Master_Protocol_synopsis_Final_18022020.pdf, this scale reflects a range from uninfected to dead, where 0 is ""no clinical or virological evidence of infection"", 1 is ""no limitation of activities"", 2 is ""limitation of activities"", 3 is ""hospitalized, no oxygen therapy"", 4 is ""oxygen by	c	NA	NA		j1PO1	The primary endpoint was the Day 14 COVID ordinal outcomes scale (Table E1 in the online supplement). This endpoint ranges from 1 (home without limitations on usual activities) to 8 (death).	c	NA	no discrepancy

ID		Registry entry (R)	C	p#PO #	Preprint (P)	C	j#PO#	Journal Article (J)	C	Discrepancy rating	
										R vs. P	R vs. J
		mask or nasal prongs"", 5 is ""non-invasive ventilation or high-flow oxygen"", 6 is ""intubation and mechanical ventilation"", 7 is ""ventilation + additional organ support - pressors, RRT (renal replacement therapy), ECMO (extracorporeal membrane oxygenation)"", and 8 is ""death""."									
64	rPO1	Survival [Time Frame: Day 21] The primary endpoint of this trial is the survival on day 21. The primary endpoint, as a dichotomous composite of survival (yes/no) and no longer fulfilling criteria of severe COVID-19, will be analyzed according their classification. Specifically, categorical variables will be analyzed by means of absolute and relative frequencies, and all continuous variables will be described using arithmetic mean, standard deviation, median, quartiles. Also, geometric means, variance and 95% confidence intervals (CI), will be calculated for all pharmacokinetics parameters.	c	p1PO1	The primary endpoint was survival on day 28.	c	NA	NA		Change in PO definition	NA
	rPO2	Survival [Time Frame: Day 35]The primary endpoint of this trial is the survival on day 35.	c	NA	NA		NA	NA		PO omitted	NA
	rPO3	Survival [Time Frame: Day 60]The primary endpoint of this trial is the survival on day 60.	c	NA	NA		NA	NA		PO omitted	NA
65	rPO1	Incidence confirmed case of COVID-19 [Time Frame: 3 weeks] Participant with RTPCR positive for SARS-CoV-2 and with or without symptoms will be	c	NA	NA		j1PO1	The primary outcome of this study was the incidence of COVID- 19 (definite [...]) among the participants. [...] Participants were followed up for 4 weeks by telephone or	c	NA	Change in PO definition

ID		Registry entry (R)	C	p#PO#	Preprint (P)	C	j#PO#	Journal Article (J)	C	Discrepancy rating	
										R vs. P	R vs. J
		defined as definite COVID-19 case.						physically as and when required.			
	rPO2	Incidence of probable case of COVID-19 [Time Frame: 3 weeks] The participant with new onset symptoms, but RTPCR negative for SARS-CoV-2 or could not be performed for any reason will be defined as probable COVID-19 case.	c	NA	NA		j1PO2	The primary outcome of this study was the incidence of COVID- 19 ([...] probable) among the participants. [...] Participants were followed up for 4 weeks by telephone or physically as and when required.	c	NA	Change in PO definition
66	rPO1	Number of Cured Patients [Time Frame: 4 weeks] Primary outcome is assessed by calculating the number of patients who had symptoms free and two successive readings of negative PCR swab.	c	p1PO1	The primary outcome was percentage of the cured patients within 23 days. Cure of the patients was defined by assessing proportion of patients who were symptoms free to be discharged from the hospital and included body temperature returned to normal for longer than 3 days, respiratory symptoms significantly improved, and 2 consecutive negative PCR test results from nasopharyngeal swabs at least 24 hours apart.	c	NA	NA		Change in PO definition	NA
67	rPO1	Clinical recovery (composite) within 14 days from initiation of study treatment until normalization of fever (≤37.2 °C oral), respiratory rate (≤24/minute on room air), and oxygen saturation (≥94% on room air), sustained for at least 24 hours; Method of measurement: Clinical examination; daily up to 14 days after starting the trial	c	NA	NA		j1PO1	The primary endpoint of this trial was clinical recovery within 14 days of enrolment. Clinical recovery was defined as normalization of fever (≤37.2°C), respiratory rate (≤24/min) and oxygen saturation (≥94%) without supplementary oxygen therapy sustained for at least 24 h. If patients maintained these criteria for over 24 h they were safely discharged from hospital.	c	NA	no discrepancy
68	rPO1	Percentage of people improved with arbidol (follow up with phone); 5, 7 and 14 days after taking Arbidol	ic	p1PO1	Clinical symptoms of patients with COVID-19 infection, such as nausea, vomiting, diarrhea, cough, shortness of breath, fever, body aches, loss of	ic	NA	NA		NA	NA

ID		Registry entry (R)	C	p#PO#	Preprint (P)	C	j#PO#	Journal Article (J)	C	Discrepancy rating	
										R vs. P	R vs. J
					appetite, and other symptoms were also monitored, and changes in symptoms in terms of improvement or worsening were reported on the 3 rd day as primary outcome						
69	rPO1	Plasma cytokine levels after administration of Qing Fei Pai Du Tang for 3 days	c	p1PO0	none defined	nd	NA	NA		no PO defined	NA
70	rPO1	Plasma cytokine levels 1 hour after the administration of JHQG	c	p1PO0	none defined	nd	NA	NA		no PO defined	NA
71	rPO1	Mortality on discharge from the ICU (Proportion of patients who die, according to their causes); Measurement time: during the stay in the Intensive Care Unit.	c	p1PO0	none defined	nd	j1PO0	none defined	nd	no PO defined	no PO defined
72	rPO1	Reduction of the time to the negativization of the coronavirus detection test (time to the negative of the coronavirus detection test by PCR-RT in the nasopharynx); Measurement time: 48 hours and on the 7th day after starting the treatment.	c	p1PO0	none defined	nd	NA	NA		no PO defined	NA
73	rPO1	Rate of decline in SARS-CoV-2 viral load [Time Frame: Baseline (at randomization) and at 96 hours] Viral load assessed by real time polymerase chain reaction in oropharyngeal samples	c	p1PO1	The primary outcome was rate of decline in SARS-CoV-2 viral load in the oropharynx from baseline through the rst 96 hours after randomization, using a single batch of swabs and a standardized sampling procedure to saturate them. [..] SARS-CoV-2 detection was performed in duplicate by RT-qPCR	c	j1PO1	The primary outcome was rate of decline in SARS-CoV-2 viral load in the oropharynx from baseline through the first 96 h after randomization, using a single batch of swabs and a standardized sampling procedure to saturate them. […] SARSCoV-2 detection was performed in duplicate by RT-qPCR	c	no discrepancy	no discrepancy
74	rPO1	Clinical Response [Time Frame: Assessed for the 24 hour period after tocilizumab administration] Tmax Response: Resolution of fever (from Tmax > 38C in 24H period to Tmax < 38C in	c	p1PO1	The primary clinical outcome was resolution of fever in the 24-hour period following tocilizumab, defied as a maximum temperature (Tmax24hrs) < 38·0°C.	c	NA	NA		no discrepancy	NA

ID		Registry entry (R)	C	p#PO#	Preprint (P)	C	j#PO#	Journal Article (J)	C	Discrepancy rating	
										R vs. P	R vs. J
		following 24H period, with Tmax measured by commonly accepted clinical methods [forehead, tympanic, oral, axillary, rectal]). Maximum temperature within 24-hour period of time (0:00-23:59) on the day prior to, day of, and every 24 hours after tocilizumab administration. The primary endpoint is absence of Tmax greater than or equal to 38ºC in the 24-hour period following tocilizumab administration.									
	rPO2	Biochemical Response [Time Frame: Assessed every 24 hours during patient's hospitalization, up to 4 weeks after tocilizumab administration] CRP normalization rate: Calculated as the ratio of the number of patients who achieve normal CRP value following tocilizumab administration and total number of patients who receive tocilizumab. Time to CRP normalization: Calculated as the number of hours between tocilizumab administration and first normal CRP value. CRP Response: Defined as at least 25% decrease in CRP from baseline at least 16 hours after administration of drug.	c	p1PO2	Originally, the primary biochemical outcomes were rate of and time to CRP normalization, guided by earlier tocilizumab-related work. During the conduct of the study, it became apparent that a patient could be safely discharged before CRP had normalized, making CRP normalization impractical. We therefore report the percentage of patients who achieved biochemical response, defined as a CRP reduction ≥25% from baseline in the 24-48 hours after tocilizumab administration, consistent with a decline determined by CRP half-life.	c	NA	NA		no discrepancy	NA
75	rPO1	Clinical status of subject at day 15 (on a 7-point ordinal scale): 1. Not hospitalized, no limitations on activities; 2. Not hospitalized, limitation on activities; 3. Hospitalized, not requiring supplemental oxygen; 4. Hospitalized, requiring supplemental oxygen; 5. Hospitalized, on non-invasive	c	p1PO1	The primary outcome was defined as cumulative clinical status on day 15. This endpoint consists of the sum of daily clinical status scores on the 7-point WHO ordinal scale from day 1 to 15 included. The WHO ordinal scale consists of the following 7 categories: 1) not hospitalized, no limitations on	c	NA	NA		no discrepancy	NA

ID		Registry entry (R)	C	p#PO#	Preprint (P)	C	j#PO#	Journal Article (J)	C	Discrepancy rating	
										R vs. P	R vs. J
		ventilation or high flow oxygen devices; 6. Hospitalized, on invasive mechanical ventilation or ECMO; 7. Death.			activities; 2) not hospitalized, limitations on activities; 3) hospitalized, not requiring supplemental oxygen; 4) hospitalized, requiring supplemental oxygen; 5) hospitalized, on non-invasive ventilation or high flow oxygen devices; 6) hospitalized, requiring extracorporeal membrane oxygenation (ECMO) or invasive mechanical ventilation; and 7) death.						
76	rPO1	Proportion of patients remaining free of mechanical ventilation in both groups [Time Frame: Day 7]	c	p1PO1	The primary outcome measure was the proportion of patients remaining free of mechanical ventilation in both groups on day seven.	c	NA	NA		no discrepancy	NA
77	rPO1	Change in lesion proportion (%) of full lung volume from baseline to day 28. [Time Frame: Day 28] Evaluation of Pneumonia Improvement	c	p1PO1	The primary outcome was gauged as a change in the total lesion proportion (%) of the whole lung volume from baseline to day 28, as measured by chest CT. It was defined as (total lesion proportion of the whole lung volume at day 28–total lesion proportion of the whole lung volume at baseline) / total lesion proportion of the whole lung volume at baseline.	c	NA	NA		no discrepancy	NA
				p2PO1	The primary outcome was a change in the total lesion proportion (%) of the whole lung volume from baseline to day 28, as measured using chest CT.	c	NA	NA		no discrepancy	NA
78	rPO1	Study 1- Clinical and virological outcome in exposed contacts [Time Frame: Up to 14 days after start of treatment] Incidence of secondary PCR confirmed symptomatic Covid-19 episodes among	c	p1PO1	The primary outcome was the onset of a confirmed Covid-19 episode, defined as symptomatic illness (at least one of the following symptoms: fever, cough, difficulty breathing, myalgia, headache,	c	NA	NA		no discrepancy	NA

ID		Registry entry (R)	C	p#PO #	Preprint (P)	C	j#PO#	Journal Article (J)	C	Discrepancy rating	
										R vs. P	R vs. J
		contacts after high risk PCR+ exposure			sore throat, new olfactory and taste disorder(s), or diarrhea) and a positive SARS-CoV-2 RT-PCR test. The primary outcome was assessed in all asymptomatic individuals, irrespective of the PCR result; in a post hoc analysis, we explored the outcome in individuals with positive and negative PCR separately. Time-to-event was defined as the number of days from the date of randomization/exposure to the confirmed date of the onset of symptomatic illness; [abstract]: The primary outcome was PCR-confirmed symptomatic Covid-19 within 14 days.						
	rPO2	Study 1- Transmission of SARS-CoV-2 in exposed contacts [Time Frame: Up to 14 days after start of treatment] Incidence of symptomatically compatible or a PCR-positive result regardless of symptoms	c	NA	*[The secondary outcome was the incidence of SARS-CoV-2 infection, defined as either the RT-PCR detection of SARS-CoV-2 in a nasopharyngeal specimen or the presence of any of the aforementioned symptoms compatible with Covid-19.]*		NA	NA		PO downgraded to secondary	NA
	rPO3	Study 2- Virological outcome in index cases [Time Frame: Up to 7 days after start of treatment] Reduction of viral RNA load in nasopharyngeal swabs at days 3, and 7 after treatment start.	c	p2PO1	The primary outcome was the reduction of viral RNA load in nasopharyngeal swabs at days 3, 7, and 14 after treatment start; RT-PCR testing	c	j1PO1	The primary outcome was the reduction of viral RNA load in nasopharyngeal swabs at days 3, and 7 after treatment start; RT-PCR testing	c	Change in PO definition	no discrepancy
	rPO4	Study 2- Clinical outcome in index cases [Time Frame: Up to 28 days after start of treatment] Time from randomization to complete resolution of symptoms at an extended 28-days follow-	c	NA	*[The secondary outcomes were time from randomization to complete alleviation of symptoms at an extended 21-days follow-up]*		NA	*[The secondary outcomes were clinical progression measured by a simplified version of the WHO progression scale [17] (1, not hospitalized with or without resumption of normal activities; 2, hospitalized, requiring supplemental oxygen; 3, hospitalized, requiring invasive*		PO downgraded to secondary	PO downgraded to secondary

ID		Registry entry (R)	C	p#PO#	Preprint (P)	C	j#PO#	Journal Article (J)	C	Discrepancy rating	
										R vs. P	R vs. J
								mechanical ventilation; and 4, death), and time from randomization to complete resolution of symptoms within the 28-days follow-up period]			
79	rPO1	Number of Participants With Active COVID-19 Disease at Day 14 Among Those Who Were Asymptomatic at Baseline [Time Frame: 14 days] Number of participants at 14 days post enrollment with active COVID19 disease among those who were asymptomatic at baseline.	c	NA	NA		j1PO1	The primary outcome was prespecified as symptomatic illness confirmed by a positive molecular assay or, if testing was unavailable, Covid-19–related symptoms. We assumed that health care workers would have access to Covid-19 testing if symptomatic; however, access to testing was limited throughout the trial period. Covid-19–related symptoms were based on U.S. Council for State and Territorial Epidemiologists criteria for confirmed cases (positivity for SARS-Cov-2 on PCR assay), probable cases (the presence of cough, shortness of breath, or difficulty breathing, or the presence of two or more symptoms of fever, chills, rigors, myalgia, headache, sore throat, and new olfactory and taste disorders), and possible cases (the presence of one or more compatible symptoms, which could include diarrhea).All the participants had epidemiologic linkage, per trial eligibility criteria. Four infectious disease physicians who were unaware of the trial-group assignments reviewed symptomatic participants to generate a consensus with respect to whether their condition met the case definition; [...] Outcome data were measured within 14	c	NA	no discrepancy

ID		Registry entry (R)	C	p#PO#	Preprint (P)	C	j#PO#	Journal Article (J)	C	Discrepancy rating	
										R vs. P	R vs. J
								days after trial enrollment; The primary outcome was the incidence of either laboratory-confirmed Covid-19 or illness compatible with Covid-19 within 14 days.			
	rPO2	Change in Disease Severity Over 14 Days Among Those Who Are Symptomatic at Baseline [Time Frame: baseline and 14 days] Visual Analog Scale 0-10 score of rating overall symptom severity (0 = no symptoms; 10 = most severe)	c	NA	NA		j2PO1	The initial primary outcome was an ordinal outcome by day 14 of not hospitalized, hospitalized, or intensive care unit stay or death. [...] we modify the primary end point to the change in overall symptom severity over 14 days as longitudinally measured on a 10-point visual analogue scale.	c	NA	no discrepancy
	NA	NA		NA	NA		j3PO0	none defined	nd	NA	no PO defined
80	rPO1	Mortality rate at day 28 [Time Frame: on day 28, after randomization] Mortality rate on day 28, after randomization	c	NA	NA		j1PO1	The primary outcome was 28-day mortality.	c	NA	no discrepancy
81	rPO1	Entry into Intensive Care with invasive mechanical ventilation or death from any cause or clinical aggravation [Time Frame: two weeks from participants' allocation to study arm] Entry into Intensive Care with invasive mechanical ventilation or death from any cause or clinical aggravation documented by the finding of a PaO2 / FiO2 ratio <150mm / Hg confirmed by a second arterial blood gas (ABG) measurement within four hours	c	NA	NA		j1PO1	The primary end point was clinical worsening within 14 days since randomization, defined by the occurrence of 1 of the following events, whichever occurred first:<br>1.) Admission to ICU with mechanical ventilation<br>2.) Death from any cause<br>3.) PaO2/FIO2 ratio less than 150 mm Hg in 1 of the scheduled arterial blood gas measurements or in an emergency measurement, confirmed within 4 hours by a second examination<br>Criteria for institution of mechanical ventilation were PaO2/FIO2 ratio less than 150 mm Hg, respiratory rate greater than 30 breaths/min, signs of respiratory distress, or multiorgan failure. Secondary	c	NA	no discrepancy

ID		Registry entry (R)	C	p#PO#	Preprint (P)	C	j#PO#	Journal Article (J)	C	Discrepancy rating	
										R vs. P	R vs. J
								aims included the evaluation of the efficacy of early vs late administration of tocilizumab in admission to ICU with mechanical ventilation, mortality, and tocilizumab toxic effects.			
82	rPO1	Viral clearance [Time Frame: 14 days] Two successive negative COVID-19 PCR analysis tests 48-72 hours apart	c	p1PO1	The primary endpoints were achievement of two successive negative SARS-CoV-2 PCR analysis tests 48 hours apart by nasopharyngeal swab [...]; evaluate the SARS-CoV-2 viral clearance on days three, seven and 14 .	c	NA	NA		no discrepancy	NA
	rPO2	Clinical improvement [Time Frame: 14 days] Normal body temperature for 48 hours	c	p1PO2	The primary endpoints were [...] normalization of body temperature for 48 hours [...]; objectives were evaluation of the clinical outcomes on days three, seven and 14	c	NA	NA		no discrepancy	NA
	NA	NA		p1PO3	The primary endpoints were [...] improvement of radiological abnormalities at day 14 [...]; objectives were evaluation of the clinical outcomes on days three, seven and 14	ic	NA	NA		PO added or upgraded from non-primary	NA
	NA	NA		p1PO4	The primary endpoints were [...] and discharge rate out of the hospital	ic	NA	NA		PO added or upgraded from non-primary	NA
83	rPO1	Requirement for invasive ventilation [Time Frame: through study completion up to 28 days] Could the plasma therapy avoid or delay the need for invasive ventilation	c	p1PO1	The primary outcome was the requirement for ventilation; the primary end points were requirement for invasive or non-invasive ventilation, in patients who required ventilation, the duration of ventilation	ic	NA	NA		Change in PO definition	NA
84	rPO1	The primary endpoint is the time from administration of the investigational agent (or placebo) to requiring mechanical ventilation and	c	NA	NA		j1PO1	The primary outcome was intubation (or death, for patients who died before intubation) after administration of tocilizumab or placebo,	c	NA	no discrepancy

ID		Registry entry (R)	C	p#PO#	Preprint (P)	C	j#PO#	Journal Article (J)	C	Discrepancy rating	
										R vs. P	R vs. J
		intubation, or death for subjects who die prior to intubation [Time Frame: 28 days] The primary endpoint is the time from administration of the investigational agent (or placebo) to requiring mechanical ventilation and intubation, or death for subjects who die prior to intubation						assessed in a time-to-event analysis. [...] Data from patients who were event-free at the end of followup were censored at 28 days (for the primary and first secondary outcome)			
85	rPO1	Cumulative incidence of SAEs through day 30 [Time Frame: 30 days]	c	NA	NA		j1PO1	The primary safety outcome was the cumulative incidence of serious adverse events (SAEs) [...]; at day 30	c	NA	no discrepancy
	rPO2	Cumulative incidence of grade 3 or 4 AEs through day 30 [Time Frame: 30 days]	c	NA	NA		j1PO2	The primary safety outcome was the cumulative incidence of [...] grade 3 or 4 adverse events [...] at day 30; at day 30	c	NA	no discrepancy
	rPO3	Incidence of discontinuation of therapy (for any reason) [Time Frame: 30 days]	c	NA	NA		j1PO3	The primary safety outcome was the cumulative incidence of [...] and/or discontinuation of therapy at day 30.; at day 30	c	NA	no discrepancy
	rPO4	Severe disease progression composite outcome [Time Frame: 14 days] Including any of the following: mortality, ICU admission, invasive mechanical ventilation, ECMO, and/or hypotension requiring vasopressor support by the 14-day post-treatment evaluation (PTE)	c	NA	NA		j1PO4	The primary efficacy outcome was the proportion of subjects meeting a severe COVID-19 progression composite end point (death, ICU admission, mechanical ventilation, ECMO, and/or vasopressor use) at day 14.	c	NA	no discrepancy
86	rPO1	Death versus survival of treated patients [Time Frame: Up to 8 weeks] evaluate the role of convalescent plasma in saving life of treated patients by measuring the final outcome whether treated patients survived or died	c	p1PO0	none defined	nd	j1PO0	none defined	nd	no PO defined	no PO defined
87	rPO1	All-cause mortality [Time Frame: Within 28 days after randomisation] For each pairwise comparison with the	c	p1PO1	The primary outcome was all-cause mortality within 28 days of randomization.	c	j1PO1	The primary outcome was all-cause mortality within 28 days after randomization;	c	no discrepancy	no discrepancy

ID		Registry entry (R)	C	p#PO#	Preprint (P)	C	j#PO#	Journal Article (J)	C	Discrepancy rating	
										R vs. P	R vs. J
		'no additional treatment' arm, the primary objective is to provide reliable estimates of the effect of study treatments on all-cause mortality.						further analyses were specified at 6 months.			
				p2PO1	Outcomes were assessed at 28 days after randomisation, with further analyses specified at 6 months. The primary outcome was all-cause mortality.	c	NA	NA		no discrepancy	NA
				NA	NA		j2PO1	Outcomes were assessed at 28 days after randomisation, with further analyses specified at 6 months. The primary outcome was 28-day all-cause mortality	c	NA	no discrepancy
				NA	NA		j3PO1	The primary outcome was all-cause mortality within 28 days after randomization; further analyses were specified at 6 months.	c	NA	no discrepancy

Note. R = registry entry, P = preprint, J = journal article; C = completeness of primary outcome, c = complete, ic = incomplete; NA = not applicable; PO = primary outcome; p#PO# = sequential number or preprint per trial and sequential number of primary outcome per preprint; j#PO# = sequential number of journal article per trial and sequential number of primary outcome per journal article. References for each entry can be found in Additional file 2.
